# Anharmonicity and Spectra–Structure Correlations in MIR and NIR Spectra of Crystalline Menadione (Vitamin K_3_)

**DOI:** 10.3390/molecules26226779

**Published:** 2021-11-10

**Authors:** Krzysztof B. Beć, Justyna Grabska, Christian W. Huck, Sylwester Mazurek, Mirosław A. Czarnecki

**Affiliations:** 1 CCB-Center for Chemistry and Biomedicine, Institute of Analytical Chemistry and Radiochemistry, Leopold-Franzens University, 6020 Innsbruck, Austria; Justyna.Grabska@uibk.ac.at (J.G.); Christian.W.Huck@uibk.ac.at (C.W.H.); 2 Faculty of Chemistry, University of Wrocław, 50-383 Wrocław, Poland; sylwester.mazurek@chem.uni.wroc.pl

**Keywords:** near-infrared (NIR), mid-infrared (MIR) spectroscopy, overtones, combination bands, anharmonicity, periodic boundary system, menadione, vitamin K_3_

## Abstract

Mid-infrared (MIR) and near-infrared (NIR) spectra of crystalline menadione (vitamin K_3_) were measured and analyzed with aid of quantum chemical calculations. The calculations were carried out using the harmonic approach for the periodic model of crystal lattice and the anharmonic DVPT2 calculations applied for the single molecule model. The theoretical spectra accurately reconstructed the experimental ones permitting for reliable assignment of the MIR and NIR bands. For the first time, a detailed analysis of the NIR spectrum of a molecular system based on a naphthoquinone moiety was performed to elucidate the relationship between the chemical structure of menadione and the origin of the overtones and combination bands. In addition, the importance of these bands during interpretation of the MIR spectrum was demonstrated. The overtones and combination bands contribute to 46.4% of the total intensity of menadione in the range of 3600–2600 cm^−1^. Evidently, these bands play a key role in shaping of the C-H stretching region of MIR spectrum. We have shown also that the spectral regions without fundamentals may provide valuable structural information. For example, the theoretical calculations reliably reconstructed numerous overtones and combination bands in the 4000–3600 and 2800–1800 cm^−1^ ranges. These results, provide a comprehensive origin of the fundamentals, overtones and combination bands in the NIR and MIR spectra of menadione, and the relationship of these spectral features with the molecular structure.

## 1. Introduction

Vitamins play an important role in living organisms, therefore are subject of numerous experimental and theoretical studies [1,2]. The investigations cover the fundamental chemical and biological processes involving vitamins, e.g., electron transfer reactions [3] as well as their biochemical activity in living organisms [4]. However, equally important are studies on the physical and chemical properties of vitamins. Different experimental methods were used to examine the correlation between the properties and the biochemical functions of vitamins, e.g., interactions of vitamins with the chemical environment and solubility in various solvents [5,6] their antioxidant potentials [7] photosensitivity and photostability [8] and the relationships between their molecular structure and chemical stability [9].

Among numerous experimental methods, vibrational spectroscopy is particularly powerful tool since it provides detailed information on changes at a molecular level. Fundamental vibrations of vitamins were intensively examined by various spectroscopic techniques, i.e., mid-infrared (MIR; i.e., infrared, IR), far-infrared (FIR), terahertz (THz) and Raman. For example, attenuated total reflectance terahertz (ATR-THz) spectroscopy of low-lying vibrational modes provided information on the effect of temperature on the structure of vitamin C [10]. MIR spectroscopy is also useful in studies of different aspects of intermolecular interactions of vitamins [11,12]. Vitamins were also examined by more sophisticated spectroscopic techniques, such as surface-enhanced IR spectroscopy (SEIRA), which provided information on temperature-induced changes in structure of vitamin B_3_ absorbed at metal surface [13]. Another example is an application of the resonance Raman for studies of unique insight into structural sensitivity of vitamin B_12_ [14].

Interpretation of the vibrational spectra is based on the characteristic group frequencies that are often doubtful. For example, assignment of the fundamental bands in IR, Raman and SERS spectra of vitamin C was discussed until recently [15]. Thus, particular interest has been paid for application of the computational methods to aid interpretation of the spectra of vitamins. The theoretical vibrational analysis provided assignments of the fundamental bands in MIR and Raman spectra of vitamin C [16,17], including the anion and cation forms [18]. The other Density Functional Theory (DFT) calculations elucidated the contribution of tautomeric forms of vitamin C to the experimental ultraviolet-visible (UV-VIS), nuclear magnetic resonance (NMR) and MIR spectra [19]. Theoretical calculations were often used to study the impact of hydrogen bonding on the molecular structure of vitamin C [20]. Additionally, the changes in the vibrational spectra resulting from interaction between vitamin C and water were investigated [21]. Recent examination of the hydrogen bonding in the crystalline phase by Car-Parinello molecular dynamics allowed to observe the double minimum proton potentials [22]. On the other hand, exploration of the vibrational spectra of other vitamins by computational chemistry were occasional. For example, the vibrational spectra of vitamin K were subject of few theoretical studies [23,24,25,26]. One has to mention the quantum chemical examinations of molecular structure and the MIR and Raman spectra of vitamin B_3_ [27,28].

In contrast, the studies of the spectra–structure relationships in near-infrared (NIR) range are rare. First of all, it results from higher complexity of NIR spectra as compared with IR or Raman ones [29,30,31]. As a result, no successful investigations of the overtones and combination bands of vitamins have been reported so far. Therefore, the understanding of NIR spectra of vitamins is poor, despite of using of two-dimensional correlation spectroscopy (2DCOS), which enhances the spectral and structural selectivity of NIR spectra [32].

Recent progress in the hardware and new theoretical methods allows the calculations of NIR spectra and improve their interpretability [29,30,33]. This gives an opportunity to explore NIR spectra of vitamins and their relationships with the IR ones. NIR spectroscopy is often applied for determination of vitamin content in fruits and extracts [34,35], pharmaceutical formulations [36] or powdered mixtures and solutions [37]. However, these analytical applications of NIR spectroscopy do not rely on interpretation of the characteristic vibrational frequencies. In this work we undertook an experimental and theoretical investigation of vibrational spectra of menadione (vitamin K_3_). Menadione is of great importance for life since it is responsible for proper blood clotting [38]. The molecule of menadione possess naphthoquinone unit that never has been examined by NIR spectroscopy. Further, the presence of C=O group in molecule of menadione provides an opportunity for comparison with the spectral characteristic of the same group in ketones [39]. This work is focused on the NIR spectrum, however, manifestation of the overtones and combination bands in the MIR spectra will be shown as well. Despite importance of non-fundamental transitions, the analysis of these bands in the MIR spectra has been occasional [40,41,42,43].

To increase the reliability of the spectra-structure correlations, the experimental spectra of vitamin K_3_ were measured in a well-defined crystalline state. The interpretation and discussion of the fundamental bands is based on the periodic DFT calculations (i.e., infinite models within periodic boundary conditions) of MIR spectra, which represent a substantial advancement over previous studies based on the finite models [17]. The cost of currently available implementation of the anharmonic calculations for periodic systems do not permit for application of a similar theoretical approach to NIR spectra. Therefore, NIR spectra were simulated using a single-molecule with the geometry extracted from the crystalline structure and then optimized in vacuum. As demonstrated recently, such approach is feasible due to local character of the vibrational modes in the NIR region [33,44]. Numerous studies have been devoted to investigations of hydrogen bonding effects in MIR spectra [20,22,45,46,47]. Recently, increases the interest in studying weak interactions, such as CH···O hydrogen bonding [48,49]. However, the investigations of these interactions in NIR spectra have been started not long ago [44,50,51,52,53,54]. The CH···O hydrogen bonding is very weak, and therefore difficult to observe in MIR spectra. On the other hand, electrical anharmonicity leads to increased intensity of NIR bands of X-H group involved in weak hydrogen bonding [55,56]. The first overtone of the carbonyl group is located in MIR region, while the second overtone appears in NIR region [39]. Therefore, simultaneous examination of MIR and NIR spectra of menadione in a well-defined crystalline phase is expected to yield helpful information on the CH···O=R interactions in vibrational spectra.

The aim of this work was providing a detailed assignment of all fundamental bands as well as overtones and combination bands of menadione in the crystalline state. On the basis of these assignments, we elucidated the spectra–structure correlation in MIR and NIR regions. These correlations give an opportunity for study of the structure and interactions of menadione in different environments. Besides, information included in this work has a great potential in analytical applications.

## 2. Results and Discussion

### 2.1. Comparison of Experimental and Theoretical (Harmonic Approximation within Periodic Model) MIR Spectra

Comparing the experimental ATR-IR and DRIFT (diffuse reflectance infrared Fourier transform) spectra one can notice obvious differences resulting from the specificity of each method (Figures S1 and S2 in Supplementary Materials). Firstly, the measured spectral intensity in DRIFT spectrum is close to optimal, as the concentration of menadione could be easily adjusted by mixing with KBr. Furthermore, the penetration depth in ATR method depends on optical properties of the sample and ATR crystal and is reduced towards the shorter wavelengths (i.e., higher wavenumbers). This effect is easily seen in the normalized spectra (Figure S2). As a result, the spectral features in the high frequency region of ATR-IR spectrum (4000–1800 cm^−1^) are very weak. In contrast, this spectral region is much better developed in the DRIFT spectrum. Therefore, we used DRIFT spectrum of menadione for further discussion.

Regardless of the method used (i.e., B3LYP/”Gatti” or B3LYP/TZVP), the MIR spectra in the 3400–2800 cm^−1^ region (Figure S3) are poorly reconstructed by harmonic calculations and numerous bands are missing. Evidently, the contributions from the anharmonic vibrations (overtones and combination bands) in this spectral region are significant, and therefore, harmonic spectra calculated by periodic B3LYP/”Gatti” and B3LYP/TZVP methods do not provide basis for reliable bands assignments.

**Figure 1:**
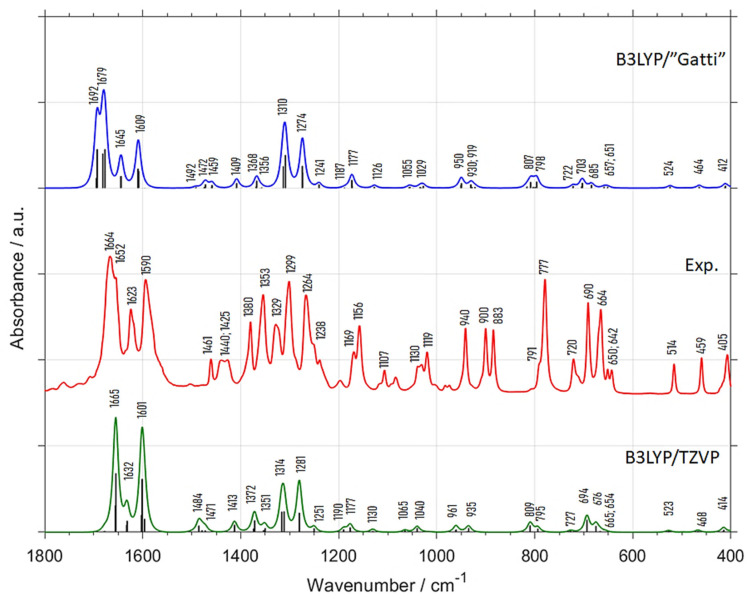
Spectrum of crystalline menadione in the 1800–400 cm^−1^ range together with the harmonic spectra calculated by periodic DFT (B3LYP/”Gatti” and B3LYP/TZVP). The detailed band assignments are presented in Table 1.

In contrast, the harmonic spectra in the 1800–400 cm^−1^ range (Figure 1) obtained by B3LYP/”Gatti” and B3LYP/TZVP methods are similar to each other, and resemble the DRIFT spectra. In spite of some differences in the peak positions and relative intensities both simulated spectra develop the same spectral features. This means that the anharmonic modes have minor contribution in this spectral range. Hence, one can conclude that the fundamental bands observed in IR spectrum of crystalline menadione can be reliably identified by the periodic harmonic calculations (Figure 1 and Table 1). In addition, we briefly investigated whether any spectral changes appear upon increasing the sample temperature from room temperature to the temperature of ca. 363 K (Figure S4 in Supplementary Materials), below melting point of menadione at 380 K. From Figure S4 it is evident that the spectra recorded at room and elevated temperatures are similar, indicating that the crystalline structure of menadione is very stable and dominates in the solid sample. Therefore, the crystalline model considered in this work is representative for the wider range of experimental conditions.

### 2.2. Overtones and Combination Bands in MIR Spectrum of Menadione

**Figure 2:**
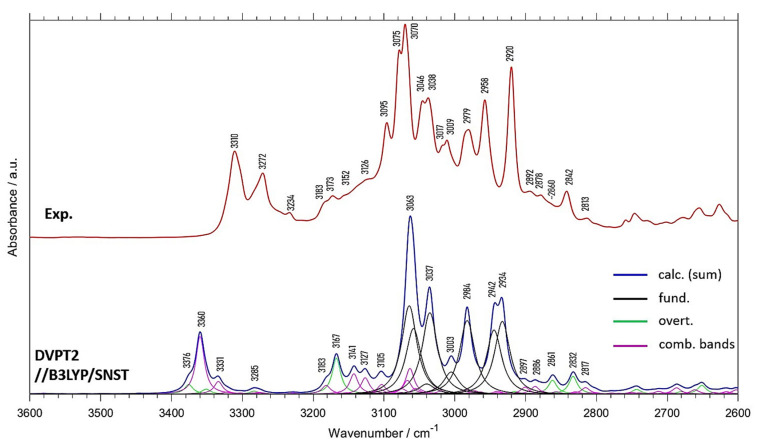
Spectrum of crystalline menadione in the 3600–2600 cm^−1^ range together with the anharmonic vibrational spectra (DVPT2//B3LYP/SNST). The detailed band assignments are presented in Table 2.

Figure 2 and Table 2 reveal that much better reconstruction of the spectra of menadione in the 3600–2600 cm^−1^ region was obtained by anharmonic calculations. It is of note that only relatively narrow spectral range (3100–2900 cm^−1^) originates from the fundamental transitions, while outside this range dominate the combination and overtone modes (Figure 2). To obtain quantitative information on the contributions from different vibrational modes, we calculated the integral intensities of the theoretical anharmonic spectrum in the 3600–2600 cm^−1^ region. It is of note that the fundamental bands contribute only to 53.6% of the total integral intensity in this region, while the contributions from the combination and overtone bands are 34.8% and 11.6%, respectively. This illustrates very well the importance of the non-fundamental transitions in this fragment of the spectrum of menadione. The distinct peaks at 3310 and 3272 cm^−1^, as well as broader absorption structures observed at ca. 3183–3126 cm^−1^ and 2892–2813 cm^−1^ (including a better resolved peak at 2842 cm^−1^) originate solely from the first overtones and binary combination bands (Figure 2 and Table 2). The significant contribution from the anharmonic vibrations in this spectral region is the main reason of poor agreement between the harmonic spectra and the experimental ones (discussed in Section 2.1). This is another example of great impact of the non-fundamental peaks on the νC-H region of MIR spectrum [40]. Note, as DVPT2 calculations offer anharmonic transition intensities as well, the IR spectra calculated with this method improve the accuracy not only of the positions of the calculated fundamental bands but also their intensities, as compared with harmonic calculations.

**Figure 3:**
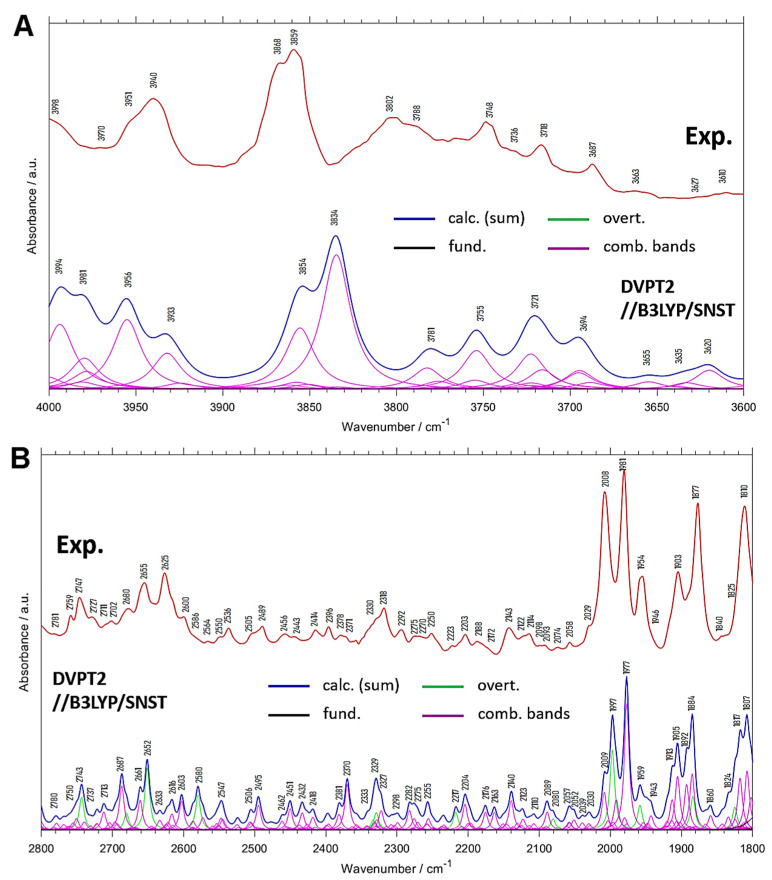
Spectrum of crystalline menadione in the 4000–3600 cm^−1^ (**A**) and 2800–1800 cm^−1^ (**B**) range together with the anharmonic (DVPT2//B3LYP/SNST) spectrum.

The presence of the first overtones and binary combination bands is clearly manifested in the regions of MIR spectra free from the fundamental bands such as 4000–3600 cm^−1^ (Figure 3A) or 2800–1800 cm^−1^ (Figure 3B). The corresponding band assignments are collected in Table 3 and Table 4. These spectral regions are often neglected during the routine studies. In principle, both regions belong to IR range, however, they include only the bands from non-fundamental transitions (overtones and combinations), just like NIR spectra. In the case of simple deuterated compounds, the range of MIR spectrum without fundamentals may cover the range from 4000 to 2300 cm^−1^ [42,43]. Figure 3B reveals that in the 2800–1800 cm^−1^ range appears a lot of peaks that may be interpreted with aid of the anharmonic calculations. It is of note that below 2000 cm^−1^ the highest frequency fundamental band is observed at 1664 cm^−1^ and it originates from a mixed δ(ring) and νC=O vibration.

The domination of anharmonic transitions is clearly seen in the 2800–1800 cm^−1^ region. The bands due to the first overtones are less numerous than the combination bands. Yet, they clearly appear in the experimental MIR spectrum of menadione, e.g., at 2747, 2655 or 2625, and 2008 cm^−1^ (Figure 3B and Table 4). In the range of 2010–1800 cm^−1^, occur few sharp peaks of relatively high intensity, originating mainly from the combination bands involving γC-H vibrations. In addition, the overtones of γC-H modes contribute to this range, but this contribution is much smaller.

### 2.3. NIR Spectrum of Menadione

**Figure 4:**
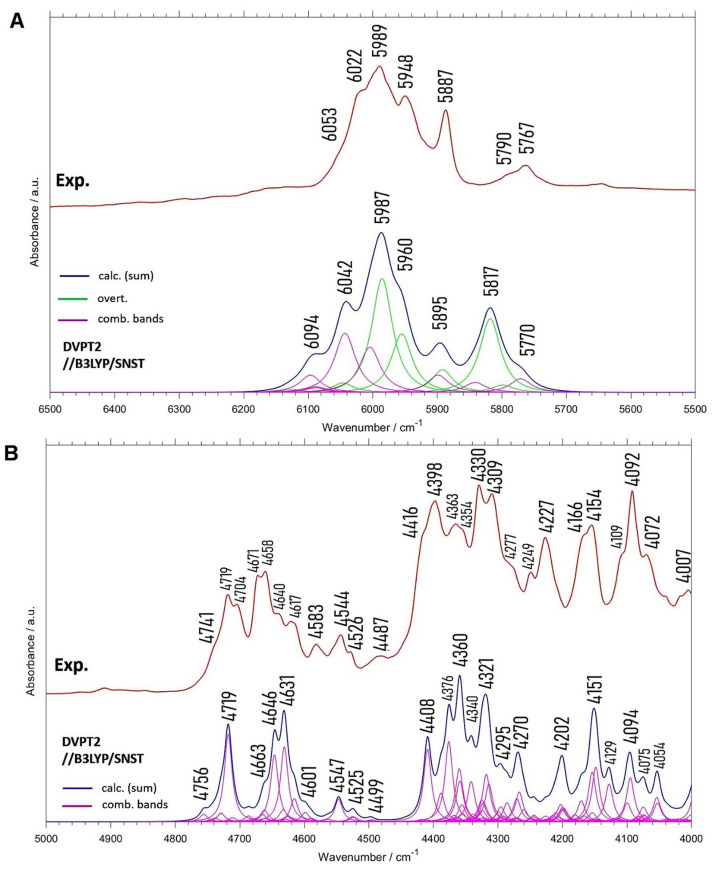
Experimental and calculated NIR spectra of Vitamin K_3_ in 6500–5000 cm^−1^ (**A**) and 5000–4500 cm^−1^ (**B**) region.

Most of bands in NIR spectrum of crystalline menadione is located in two spectral regions: 6100–5700 cm^−1^ (Figure 4A) and 4800–4100 cm^−1^ (Figure 4B). Outside these regions are observed only extremely weak bands from the higher order overtones and combination bands. To estimate the relative importance of the overtones and combination bands in the NIR range, we calculated integral intensity values for the theoretical spectrum. It appears that in the 7000–4000 cm^−1^ range the binary combinations and the first overtones contribute to 70% and 30% of total intensity, respectively. Evidently, the combination bands are the main component of the NIR spectrum of crystalline menadione.

The 6100–5700 cm^−1^ region is contributed mainly by the first overtones, while the contribution from the binary combinations is visible at 6022 cm^−1^ (Figure 4A). The other higher frequency combinations (e.g., 6094 cm^−1^) in the theoretical spectra are less clear in the experimental spectrum. Some of these bands appear as not resolved shoulders.

As shown, NIR spectra of even relatively simple molecules are complex and consist of numerous overlapped bands [57]. This feature of NIR spectra is well demonstrated in the case of crystalline menadione. In Figure 4, the experimental and calculated NIR spectra are compared. For the theoretical spectra, the contributions from all individual overtones and combination bands are also shown. Evidently, the degree of overlap of various bands is significantly higher than that observed in the MIR spectrum (Figure 1). Consequently, the structural information included in the NIR spectrum is obscured and difficult to present in the table with the individual band assignments. Therefore, to better reflect this intrinsic complexity of NIR line shape and present the assignments in a clear way, the contributions from selected vibrational modes to NIR spectra are shown in form of color maps.

**Figure 5:**
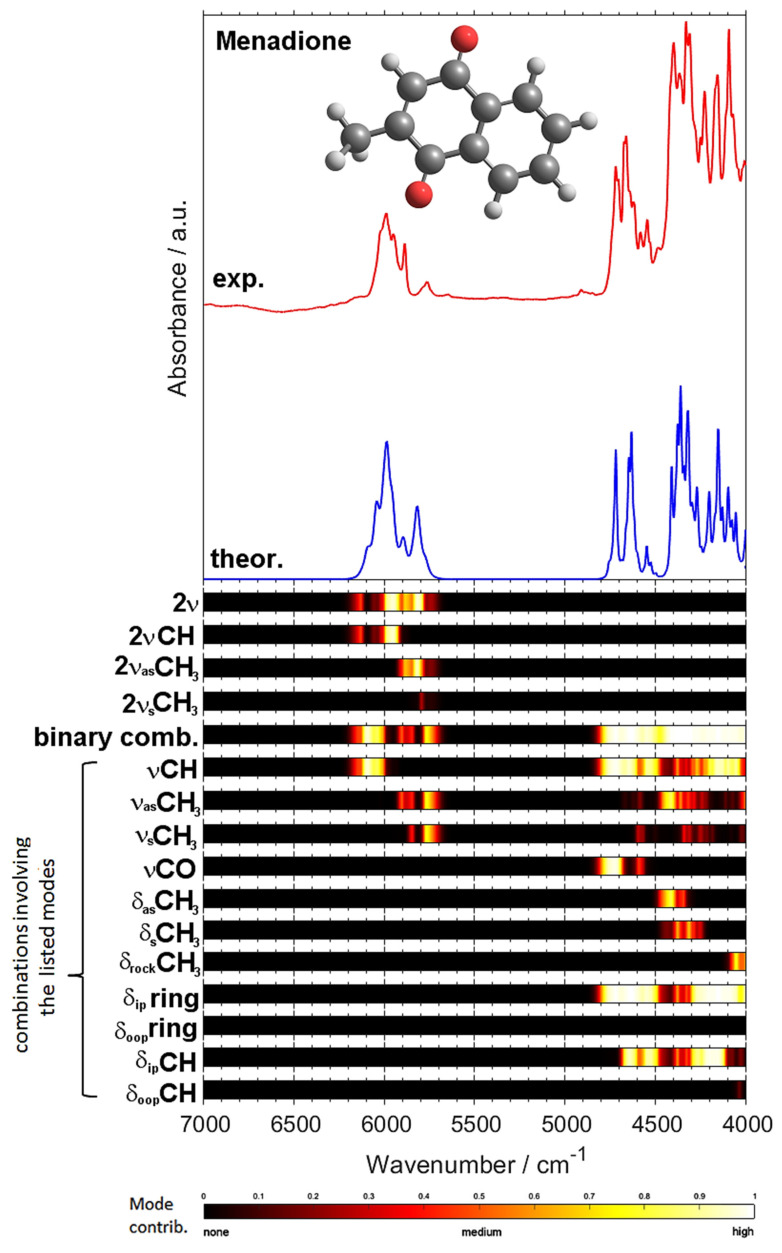
Contributions from selected vibrational modes to NIR spectrum of menadione.

As can be seen (Figure 5) most of bands contribute to 4800–4000 cm^−1^ and 6200–5700 cm^−1^ regions, confirming direct observation from the spectra. Interestingly, the extent of the overlap of peaks in the 4400–4000 cm^−1^ region is noticeable greater than that in the 4000–3600 cm^−1^ range (Figure 3A), despite similar origin of the major contributing bands, i.e., δring + νCH and δCH + νCH (see Figure 3 and Figure 5, Table 3). Obviously, the degree of overlap of NIR bands rapidly increases with the size of the molecule [40,58]. However, NIR spectrum of menadione consist of relatively sharp and well-resolved peaks, as compared with smaller molecules such as crystalline melamine [33], butyl alcohols [59] or ethanol in CCl_4_ [60,61]. Therefore, menadione offers an opportunity to elucidate reliable spectra–structure correlations in the NIR region.

As expected, the major contribution to NIR spectra originates from the first overtones of the aromatic C-H stretching modes absorbing above 5900 cm^−1^ (Figure 4A). However, the presence of the methyl group is clearly seen in the 5900–5600 cm^−1^ range. The two 2ν_as_CH_3_ bands (of different symmetry, i.e., 2ν_as_CH_3_ and 2ν_as_’CH_3_ according to Pulay’s convention [62]) appear at 5895 and 5817 cm^−1^. It seems that the former band has an underestimated intensity, and probably it corresponds to the sharp peak at 5887 cm^−1^ in the experimental spectrum. Interestingly, the 2ν_s_CH_3_ vibration has only a minor contribution at 5799 cm^−1^.

In the spectral region from 4780 to 4600 cm^−1^ appears a series of sharp peaks with a relatively small extent of overlap (Figure 4B). The pronounced absorbance at 4719 cm^−1^ corresponds to the combination band (νC=O, δring) + νCH, while the peaks at 4646 cm^−1^ and 4631 cm^−1^ are due to the other combinations (δring, δCH) + νCH. The prominent intensity of these combination bands and relatively good separation reflects strong coupling between the vibrational modes. This may result from the localization of the modes in a rigid and symmetric ring structure. Similar peaks have been recently observed in polymers having six-membered aromatic rings [63] and in NIR spectra of other systems with aromatic rings [64]. Interestingly, the combinations of ring in-plane deformation with C=O stretching vibrations are more pronounced for menadione than those for the six-membered rings [63]. Further studies are necessary to better understand the impact of the C=O bonds in the aromatic ring on presence of this characteristic pattern in NIR spectra. The presence of a series of strong and isolated combination bands in this spectral range may evidence the presence of aromatic rings in the sample.

## 3. Materials and Methods

### 3.1. Experimental

Menadione of high purity (>98%) purchased from Sigma Aldrich (St. Louis, MO, USA) was used as received. MIR spectra were measured by attenuated total reflection (ATR) and diffuse reflectance (DRIFT) techniques on a Nicolet iS50 FT-IR/NIR spectrometer (Thermo Fisher Scientific, Waltham, MA, USA) using KBr beamsplitter and DTGS/KBr detector. IR spectra were measured in the 4000–400 cm^−1^ range with a resolution of 2 cm^−1^ and 512 scans were accumulated. DRIFT spectra were measured with the Collector accessory. The sample was mixed with dry KBr to obtain approx. 2% (*w*/*w*) solid solution. The sample was prepared directly before the spectral measurements. ATR-IR spectra were acquired by ATR Smart Golden Gate accessory (single reflection, 45° incident angle) with diamond internal reflection element (IRE). Next, the spectra were subjected to ATR correction using the refractive index of the IRE and the sample. NIR spectra were measured on Nicolet iS50 spectrometer (Thermo Fisher Scientific, Waltham, MA, USA), with CaF_2_ beamsplitter and DTGS/KBr detector. The spectra were collected in the 10,000–4000 cm^−1^ range with resolution of 4 cm^−1^ and 256 scans were accumulated.

### 3.2. Computational Procedures

**Figure 6:**
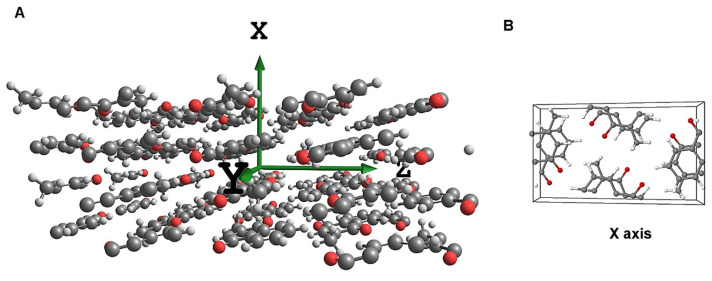
(**A**): Molecular structure of crystalline menadione used in this study; (**B**): the content of the unit cell, perspective view along the x-axis.

The simulation of MIR spectra of crystalline menadione was based on harmonic analysis in three-dimensional periodic representation of crystal structure of menadione using Crystal 09 software (Aethia Srl, Turin, Italy) [65]. An infinite 3D models of crystal lattice was constructed by defining the primitive cell based on the experimental structural data obtained from the Cambridge Structural Database (CSD) (Figure 6) [66]. At first, an unconstrained and full geometry optimization involving both the atomic centers and cell parameters was performed. The following parameters were set throughout the geometry and vibrational computing steps. The Monkhorst-Pack reciprocal space was sampled over a shrinking factor of eight. The self-consistent field (SCF) procedure was iteratively converged with a tolerance of 10^−13^ atomic units per unit cell. The truncation of Coulomb and exchange sums in direct space was controlled by setting the Gaussian overlap tolerance criteria to 10^−8^, 10^−8^, 10^−8^, 10^−8^, and 10^−16^. To accelerate the convergence of the SCF procedure, a linear mixing of Fock matrices by 25% between adjacent steps and an energy shifting of 0.8 Hartree for the first SCF cycle were employed. The electron integrals were numerically calculated over a dense (XL) integration grid. The periodic Density Functional Theory (DFT) computations were performed with the use of B3LYP (Becke, three-parameter, Lee-Yang-Parr) [67] single-hybrid density functional, as implemented in Crystal 09 software. The calculations were performed using two different basis sets. In the first one, we applied the basis sets for the respective atomic centers: 3-1p1G for hydrogen, and 6-31d1G for carbon and oxygen. As these basis sets were proposed by Gatti et al. [68], for clarity we refer them as “Gatti” basis sets. The second kind of calculations used triple-ζ valence basis set with polarization (TZVP), applied uniformly for all atomic centers (C, O, H). An application of the TZVP basis set substantially increased the computational time. Harmonic vibrational frequencies and intensities were obtained at the Gamma point in each case. The frequencies were numerically calculated by Crystal 09. To ensure the high stability of this procedure, numerical derivation (the second derivatives of the potential energy) was based on two-point finite difference scheme. The convergence criterion for the vibrational analysis was successfully achieved, as the sonic modes of the crystal lattice approached near zero values (not exceeding −0.5 cm^−1^).

Simulations of NIR spectra required multi-modal anharmonic computations in order to yield data on binary combinations, which are the most important factors in this spectral region [69]. For this purpose, we applied Deperturbed Vibrational Second-Order Perturbation Theory (DVPT2), and the calculations were performed with Gaussian 16 Rev. B.02 software [70]. Computations of NIR spectra were carried out using DFT approach at B3LYP/SNST [71] level of theory, and additionally refined by applying Grimme’s third version of empirical correction for dispersion with Becke-Johnson damping (GD3BJ) [72]. The considered system is not expected to be appreciably impacted by the dispersion forces, but the modern variants of the dispersion correction should be commonly used with DFT [73], as the gain in accuracy is achieved whenever geometry optimization is performed [74,75,76]. The accuracy of the calculated anharmonic frequencies and intensities is known to be sensitive to the precision of the optimized geometry [77]. The calculations were based on the model of a single molecule in vacuum (Figure S5 in Supplementary Materials). The initial atomic coordinates were extracted from the experimental crystalline structure and subsequently optimized to the local minimum energy. The geometry optimization was performed with very tight convergence criteria. Superfine grids were used for the evaluation of two-electron integrals and solving coupled perturbed Hartree-Fock equations.

The modelling of the MIR and NIR band shapes was carried out by using product of Lorentz-Gauss (Cauchy-Gauss) functions [51,78]. To improve the agreement between the harmonic spectra calculated in periodic conditions with the experimental MIR spectra, we applied a single-parameter scaling based on Yoshida’s wavenumber linear scaling (WLS) method [79]. Such an approach was found to yield helpful improvement of the calculated peak positions in our earlier works in which calculations of IR spectra of crystalline systems were performed [33,41]. No frequency scaling was applied to the anharmonic spectra.

## 4. Conclusions

A detailed vibrational analysis of MIR and NIR spectra of crystalline menadione (i.e., vitamin K_3_) was performed with the use of harmonic approximation applied to a periodic model of a crystal lattice and anharmonic DVPT2 calculations applied to an isolated molecule. The combination of different theoretical approaches made possible for successful reconstruction of the experimental spectra and enabled reliable band assignments. For the first time, a detailed analysis of the NIR spectrum of a molecule with naphthoquinone moiety was performed, and new spectra–structure relationships were elucidated. Our results evidence the great importance of overtones and combination bands in the interpretation of MIR spectrum of crystalline menadione. It was estimated that these non-fundamental bands contribute to 46.4% of the total integral intensity in the 3600–2600 cm^−1^ region. Evidently, they are significant factors influencing the spectral line shape. As shown, the overtones and combination bands absorbing in the regions of MIR spectrum without the fundamental bands (4000–3600 and 2800–1800 cm^−1^) provide useful structural information. Using a color map, we presented in a clear way the contribution to NIR intensity from different vibrational modes. As shown, the NIR spectrum is dominated by the combination bands, while the first overtones (from νC-H) significantly contribute only in the 6000–5800 cm^−1^ range. The total contribution to the NIR intensity from the combination modes and first overtones was estimated to be 70% and 30%, respectively. This work provides solid basis for comprehensive interpretation of NIR and MIR spectra of crystalline menadione, and establish new relationships between these two spectral regions.

## Figures and Tables

**Table 1:** Experimental and calculated band positions [cm^−1^] together with calculated relative intensities [%] and assignments of the fundamental bands in the 1800–400 cm^−1^ range for crystalline menadione.

Experimental Position (int.) ^(a)^	B3LYP/”Gatti”		B3LYP/TZVP		Position Difference (Exp—Calc)		Assignment ^(c)^
	Position	Int. ^(b)^	Position	Int. ^(b)^	B3LYP/”Gatti”	B3LYP/TZVP	
1664 (vs)	1692	82.5	1665	100.0	−28	−1	δring, νC=O
1652 (vs)	1679	100.0	-	-	−27	-	δring, νC=O
1623 (m)	1645	33.3	1632	26.5	−22	−9	δring (νCC), νC=O
1590 (s)	1609	49.1	1601	89.7	−19	−11	δring, δCH
1461 (w)	1492	1.8	1484	11.8	−31	−23	δring, δCH, δ_as_CH_3_
1440 (w)	1472	7.0	1471	5.9	−32	−31	δ_as_CH_3_
1425 (w)	1459	5.3	1413	8.8	−34	12	δ_as_CH_3_
1380 (m)	1409	8.8	1372	17.6	−29	8	δ_s_CH_3_
1353 (m)	1368	12.3	1351	8.8	−15	2	δring, δCH
1329 (m)	1356	3.5	-	-	−27	-	δring, δCH
1299 (s)	1310	66.7	1314	41.2	−11	−15	δring, δCH
1264 (m)	1274	50.9	1281	44.1	−10	−17	δring, δCH
1238 (w)	1241	5.3	1251	4.4	−3	−13	δring, δCH
1169 (w)	1187	1.8	1190	4.4	−18	−21	δring, δCH
1156 (m)	1177	1.8	1177	7.4	−21	−21	δring, δCH
1107 (vw)	1126	14.0	1130	2.9	−19	−23	δring, δCH
1030 (vw)	1055	3.5	1065	1.5	−25	−35	γCH, ρCH_3_
1019 (w)	1029	3.5	1040	7.4	−10	−21	γCH, ρCH_3_
940 (w)	950	5.3	961	5.9	−10	−21	δring
900 (w)	930	10.5	935	5.9	−30	−35	γring, γCH, ρCH_3_
883 (w)	919	7.0	-	-	−36	-	γring, γCH, ρCH_3_
791 (w)	807	12.3	809	8.8	−16	−18	γring, γCH
777 (s)	798	12.3	795	5.9	−21	−18	γring, γCH
720 (w)	722	3.5	727	1.5	−2	−7	δring
690 (m)	703	10.5	694	14.7	−13	−4	γring
664 (m)	685	5.3	676	8.8	−21	−12	γring
650 (vw)	657	1.8	665	2.9	−7	−15	δring
642 (vw)	651	1.8	654	1.5	−9	−12	δring
514 (w)	524	3.5	523	1.5	−10	−9	δring
459 (w)	464	3.5	468	1.5	−5	−9	δring
405 (w)	412	5.3	414	4.4	−7	−9	δring

^(a)^ vs—very strong; s—strong; m—medium; w—weak; vw—very weak; ^(b)^ normalized to the most intense calculated band, separately for each calculated spectrum (i.e., B3LYP/”Gatti” and B3LYP/TZVP); ^(c)^ ν—stretching; δ—in-plane bending; γ—out-of-plane bending; ρ—rocking.

**Table 2:** Experimental and calculated band positions [cm^−1^] together with calculated relative intensities [%] and assignments of the fundamental bands in the 3600–2600 cm^−1^ range for crystalline menadione. Highlighted (bold) are the fundamental bands.

Experimental Position	Exp. Intensity (Normalized)	Calc. Position	Calc. Intensity (Normalized)	Position Difference (Exp—Calc)	Assignment ^(a)^
-		3376	11.0	-	2(δring, νC=O)
3310	40.7	3360	35.1	−50	(δring, νC=O) + (δring, νC=O); 2(δring, νC=O)
3272	30.4	3331	10.2	−59	(δring, νC=O) + (δring, νC=O)
3234	11.9	3285	3.5	−51	2(δring, νC=O)
3183	16.7	3183	8.8	0	(δring, δCH) + (δring, δCH)
3173	19.8	3167	22.9	6	2(δring, δCH)
3152	19.9	3141	15.9	11	δCH + (δring, νC=O)
3126	27.7	3127	14.3	−1	δCH + (δring, νC=O)
3095	54.1	3105	12.9	−10	δCH + (δring, δCH)
3075; 3070	88.0; 100.0	3063	100.0	12; 7	νCH; δ_sym_CH_3_ + (δring, νC=O)
**3046; 3038**	**64.1; 65.6**	**3037**	**60.2**	**9; 1**	**νCH**
**3017; 3009**	**43.5; 45.8**	**3003**	**21.6**	**14; 6**	**νCH**
**2979**	**50.6**	**2984**	**49.1**	**−5**	**ν_as_CH_3_**
**2958**	**64.5**	**2942**	**51.6**	**16**	**ν_as_CH_3_**
**2920**	**79.5**	**2934**	**54.9**	**−14**	**ν_s_CH_3_**
2892	21.9	2897	8.7	−5	δring + (δring, δCH)
2878	20.2	2886	7.9	−8	(δring, δCH) + (δring, δCH)
~2860	16.1	2861	10.9	−1	2δ_asym_CH_3_
2842	21.8	2832	12.4	10	2δ_asym_CH_3_
2813	9.3	2817	6.7	−4	δ_sym_CH_3_ + δ_asym_CH_3_

^(a)^ ν—stretching; δ—in-plane bending; γ—out-of-plane bending.

**Table 3:** Experimental and calculated band positions [cm^−1^] together with calculated relative intensities [%] (normalized to the intensity of 3063 cm^−1^ band; see Table 2) and assignments of the fundamental bands in the 4000–3600 cm^−1^ range for crystalline menadione.

Experimental Position	Calc. Position	Calc. Intensity ^(a)^	Position Difference (Exp—Calc)	Assignment ^(b)^
3998	3994	1.6	4	(δring, ρCH_3_) + ν_as_CH_3_
3970	3981	1.5	−11	δring + νCH
3951	3956	1.5	−5	ρCH_3_ + ν_s_CH_3_
3940	3933	0.9	7	(δring, ρCH_3_) + ν_s_CH_3_
3868	3854	1.6	14	(δring, ρCH_3_) + νCH
3859	3834	2.5	25	(δring, ρCH_3_) + νCH
3802; 3788	3781	0.6	21; 7	δring + νCH
3748	3755	0.9	−7	δring + νCH
3718	3721	1.2	−3	δring + νCH
3687	3694	0.8	−7	δring + νCH
3663	3655	0.2	8	δring + ν_as_CH_3_
3627	3635	0.2	−8	δring + ν_as_CH_3_
3610	3620	0.4	−10	(γring, ρCH_3_) + ν_as_CH_3_

^(a)^ normalized to the most intense calculated band; ^(b)^ ν—stretching; δ—in-plane bending; γ—out-of-plane bending; ρ—rocking.

**Table 4:** Experimental and calculated band positions [cm^−1^] together with calculated relative intensities [%] (normalized to the intensity of 3063 cm^−1^ band; see Table 2) and assignments of the fundamental bands in the 2800–1800 cm^−1^ range for crystalline menadione.

Experimental Position	Calc. Position	Calc. Intensity ^(a)^	Position Difference (Exp—Calc)	Assignment ^(b)^
2781	2780	1.1	1	δ_asym_CH_3_ + (δring, δCH)
2759	2750	1.9	9	(δring, δCH) + δCH
2747	2743	3.6	4	2δ_sym_CH_3_
2727	2737	1.6	−10	(δring, δCH) + δCH
2711	2723	1.6	−12	δ_sym_CH_3_ + (δring, δCH)
2702	2713	2.1	−11	(δring, δCH) + δCH
2680	2687	4.4	−7	(δring, δCH) + (δring, δCH)
2655	2661	3.4	−6	(δring, ρCH_3_) + (δring, νC=O)
2625	2652	5.5	−27	2δring
2600	2633	1.5	−33	(δring, δCH) + (δring, δCH)
2586	2616	2.4	−30	(δring, δCH) + δring
2564	2603	2.8	−39	(δring, δCH) + (δring, δCH)
2550	2580	3.4	−30	2(δring, δCH)
2536	2547	2.3	−11	(δring, δCH) + δ_sym_CH_3_
2505	2506	1.6	−1	(δring, δCH) + (δring, δCH)
2489	2495	2.7	−6	(δring, δCH) + (δring, δCH)
2456	2462	1.2	−6	ρCH_3_ + δ_asym_CH_3_
2443	2451	2.3	−8	(δring, ρCH_3_) + δ_asym_CH_3_
2414	2432	2.2	−18	(δring, δCH) + (δring, δCH)
2396	2418	1.6	−22	ρCH_3_ + δ_sym_CH_3_
2378	2381	2.1	−3	(δring, δCH) + (δring, δCH)
2371	2370	4.1	1	(δring, δCH) + (δring, δCH)
2330	2333	4.1	−3	2(δring, δCH)
2318	2323	2.9	−5	2(δring, δCH); (δring, δCH) + (δring, δCH)
2292	2298	1.3	−6	δring (breath) + (δring, δCH)
2275	2282	2.1	−7	δring + (δring, δCH)
2270	2275	2.1	−5	(δring, δCH) + (δring, δCH)
2250	2255	2.2	−5	(δring, δCH) + (δring, δCH)
2223	2217	1.8	6	2(δring, δCH)
2203	2204	2.8	−1	δring + (δring, δCH)
2188	2176	2.3	12	δring + (δring, δCH)
2172	2163	1.8	9	δring + (δring, νC=O)
2143	2140	3.1	3	(δring, ρCH_3_) + (δring, δCH)
2111	2123	1.6	−12	(δring, ρCH_3_) + (δring, δCH)
2114	2110	1.2	4	(δring, ρCH_3_) + (δring, δCH)
2098	2089	2.3	9	(δring, ρCH_3_) + (δring, δCH)
2093	2080	1.5	13	2ρCH_3_
2074	2057	1.9	17	δring + (δring, δCH)
2058	2052	1.6	6	δring + (δring, νC=O)
2029	2030	1.4	−1	(δring, ρCH_3_) + (δring, δCH)
2008	2009; 1997	4.6/9.0	−1; 11	δring + (δring, δCH); 2γCH
1981	1977	12.0	4	γCH + γCH
1954	1959	3.5	−5	2γCH
1946	1943	2.2	3	δring + (δring, δCH)
1903	1913; 1905	5.0/6.8	−10; −2	γCH + γCH
1877	1892; 1884	6.5/9.1	−15; −7	γCH + γCH
1840	1860	1.9	−20	δring + (δring, δCH)
1825	1824	5.6	1	2γCH
1810	1817; 1807	7.8/9.0	−7; 3	γCH + γCH; δring + (δring, δCH)

^(a)^ normalized to the most intense calculated band; ^(b)^ ν—stretching; δ—in-plane bending; γ—out-of-plane bending; ρ—rocking.

## Data Availability

Data is available from the authors.

## Supplementary Materials

The following are available online, Figure S1. Untreated experimental ATR and DRIFT IR spectra of crystalline menadione in region of 4000–400 cm^−1^; Figure S2. Normalized experimental ATR-IR and DRIFT spectra of crystalline menadione in region of 4000–400 cm^−1^; Figure S3. IR (DRIFT) spectrum of crystalline menadione in the 3400–2800 cm^−1^ region together with the harmonic spectra calculated by periodic DFT (B3LYP/“Gatti” and B3LYP/TZVP); Figure S4. Experimental ATR spectra of crystalline menadione in region of 4000–400 cm^−1^ measured in room temperature (bottom) and at around 373 K. Figure S5. The molecule of menadione optimized at B3LYP-GD3BJ/SNST level of theory; Table S1. Geometrical parameters in Cartesian coordinates of menadione optimized at B3LYP-GD3BJ/SNST level of theory; Table S2. Potential energy distributions over the internal coordinates of menadione resulting from harmonic analysis performed at B3LYP-GD3BJ/SNST level of theory.

## Sample Availability

Samples of the compounds are available from the authors.
