# Supplementary material for: Anharmonicity and Spectra–Structure Correlations in MIR and NIR Spectra of Crystalline Menadione (Vitamin K3)
Source: Molecules. 2021 Nov 10;26(22):6779. doi: 10.3390/molecules26226779 (PMC8620535; doi:10.3390/molecules26226779)
Supplement: Supplementary file 1 [file molecules-26-06779-s001.zip › molecules-1441593-supplementary.pdf]

# Anharmonicity and Spectra–Structure Correlations in MIR and NIR Spectra of Crystalline Menadione (Vitamin K3)

# Supplementary Materials

**Krzysztof B. Beć [1,*], Justyna Grabska [1], Christian W. Huck [1], Sylwester Mazurek [2] and Mirosław A. Czarnecki [2,*]**

[1] CCB-Center for Chemistry and Biomedicine, Institute of Analytical Chemistry and Radiochemistry, Leopold-Franzens University, 6020 Innsbruck, Austria; Justyna.Grabska@uibk.ac.at (J.G.); Christian.W.Huck@uibk.ac.at (C.W.H.)

[2] Faculty of Chemistry, University of Wrocław, 50-383 Wrocław, Poland; sylwester.mazurek@chem.uni.wroc.pl

* Correspondence: Krzysztof.Bec@uibk.ac.at (K.B.B.); miroslaw.czarnecki@chem.uni.wroc.pl (M.A.C.)

__Figures__

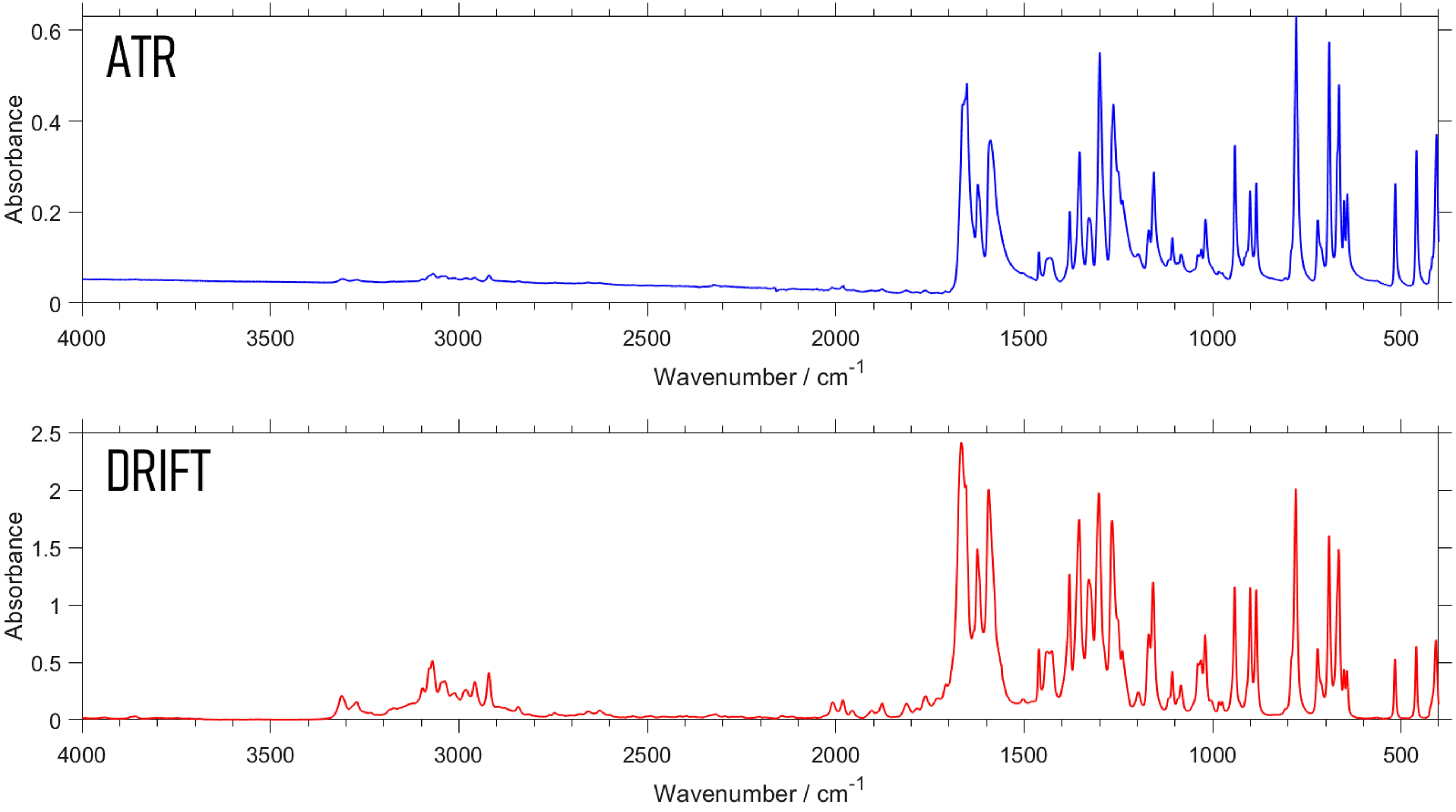


**Figure S1. Untreated experimental ATR and DRIFT IR spectra of crystalline menadione in region of 4000–400 cm⁻¹.**

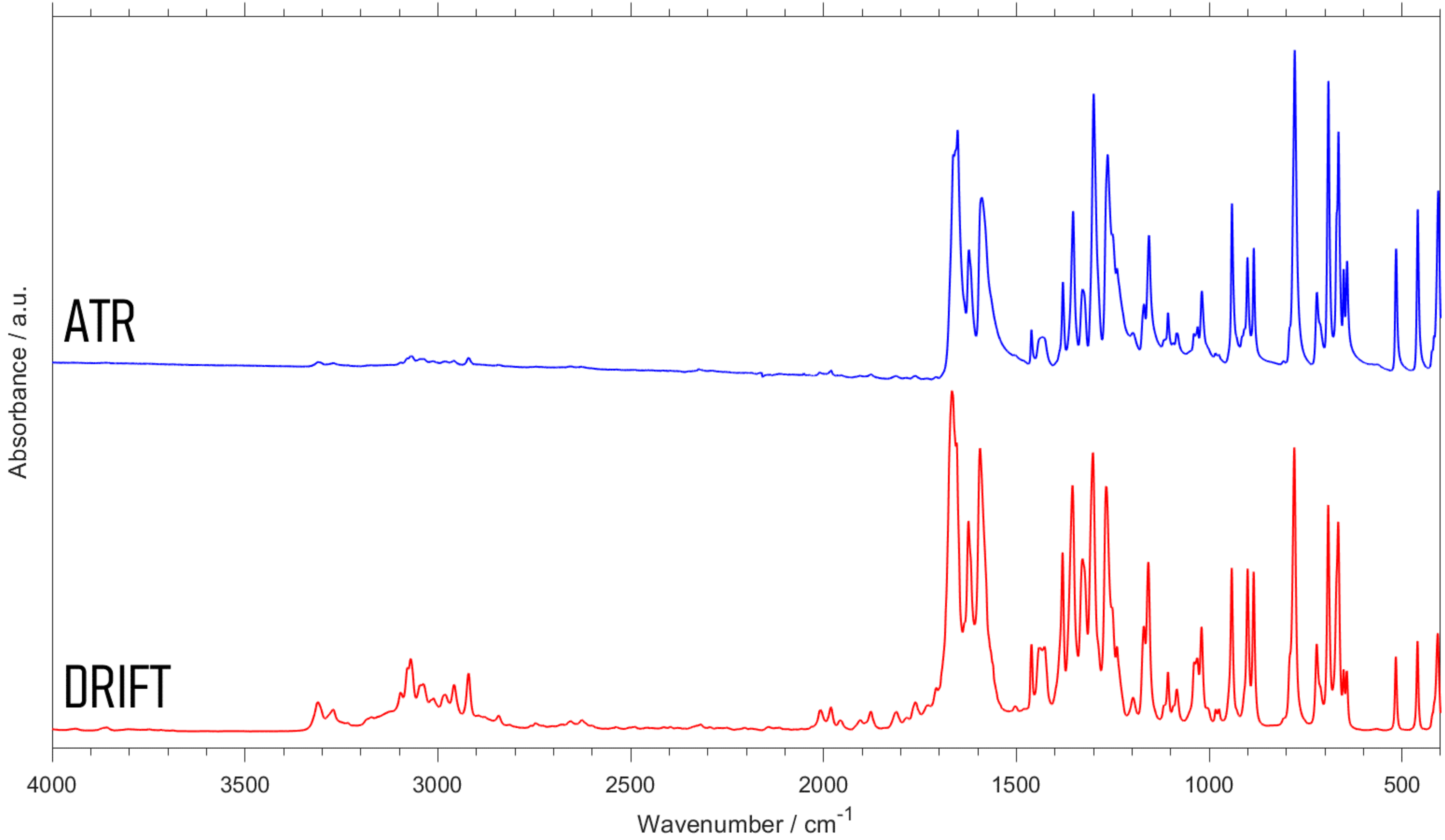


**Figure S2. Normalized experimental ATR-IR and DRIFT spectra of crystalline menadione in region of 4000–400 cm⁻¹.**

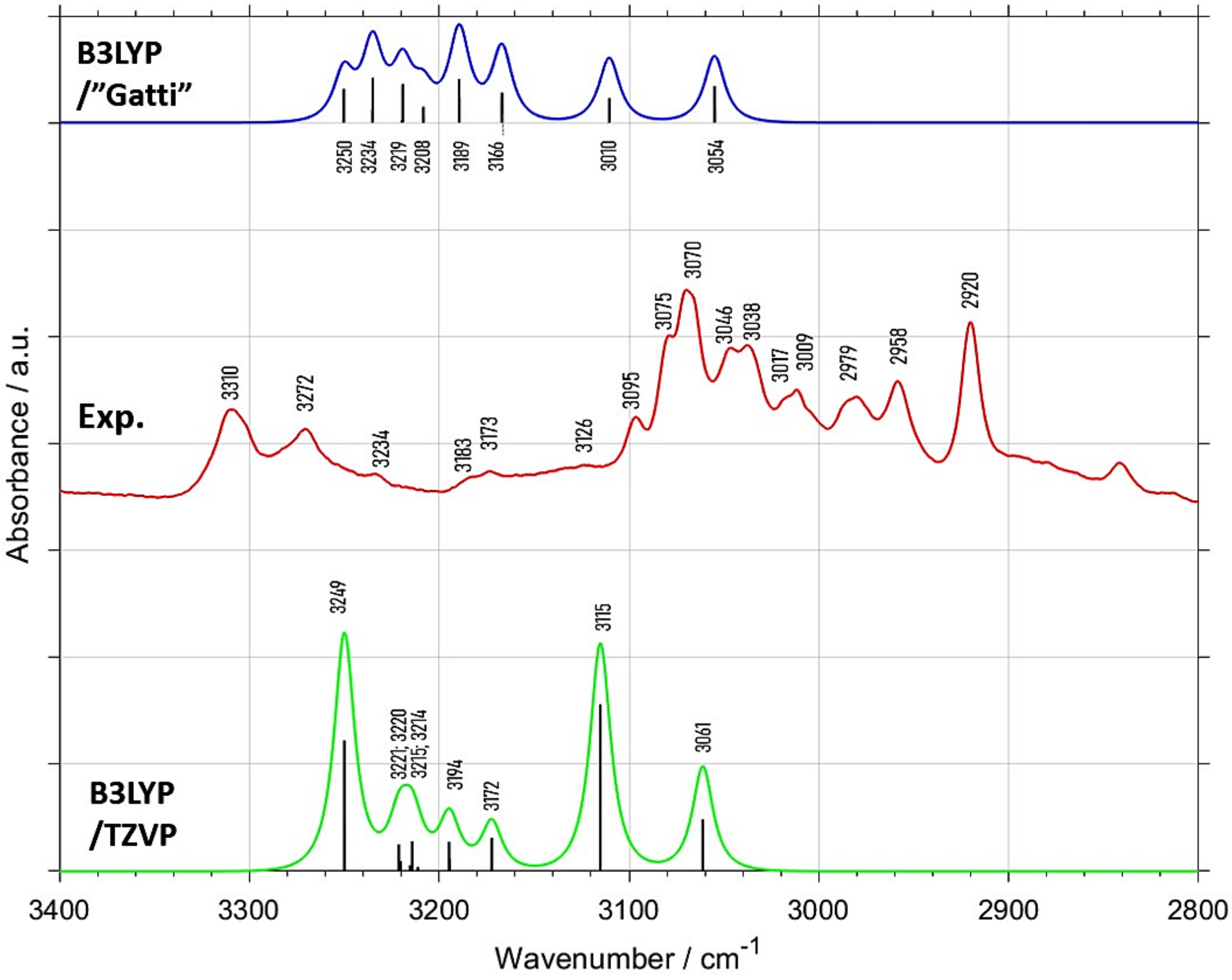


**Figure S3. IR (DRIFT) spectrum of crystalline menadione in the 3400–2800 cm$^{-1}$ region together with the harmonic spectra calculated by periodic DFT (B3LYP/"Gatti" and B3LYP/TZVP).**

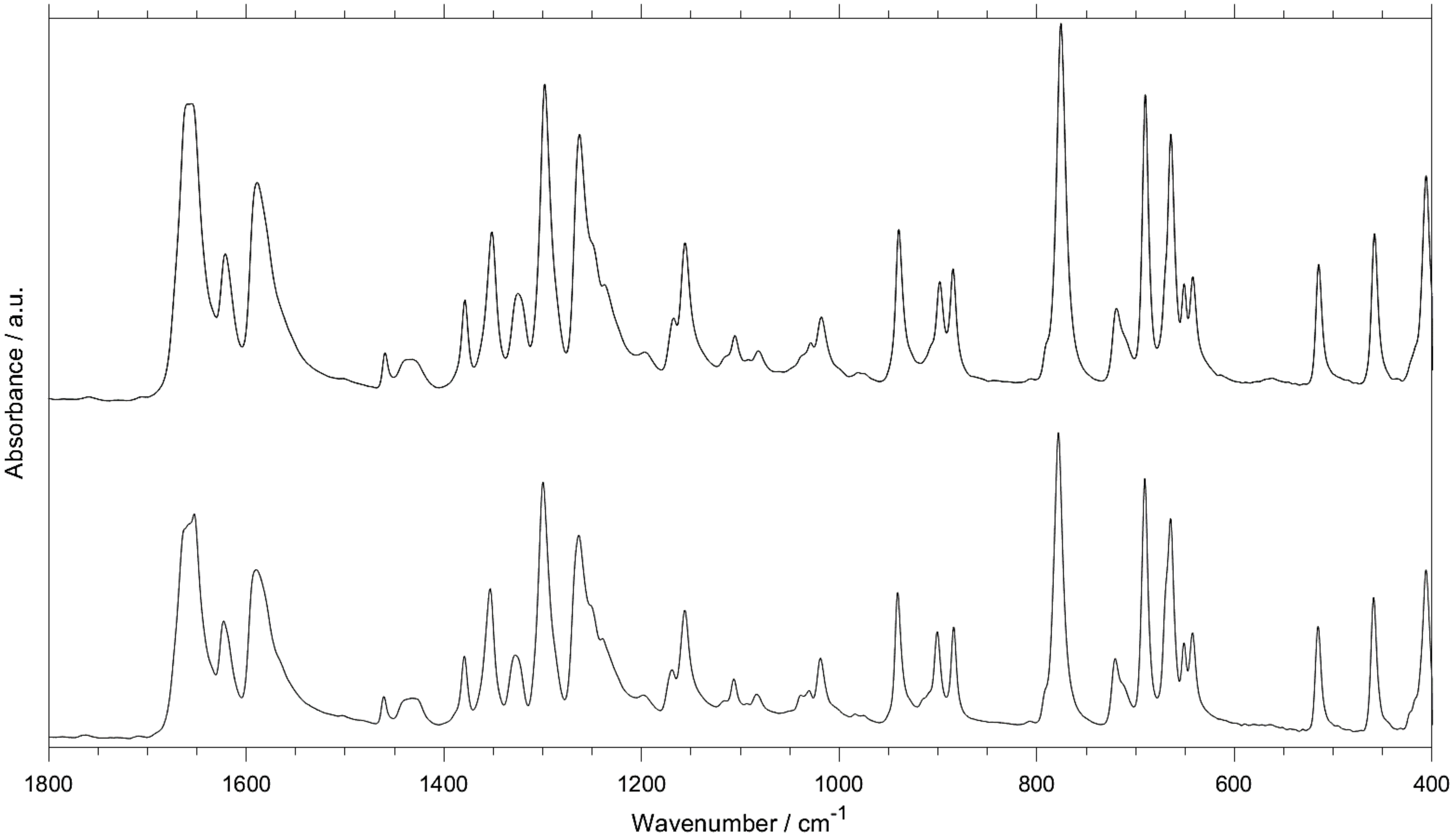


**Figure S4. Experimental ATR spectra of crystalline menadione in region of 4000–400 cm⁻¹ measured in room temperature (bottom) and at around 363 K.**

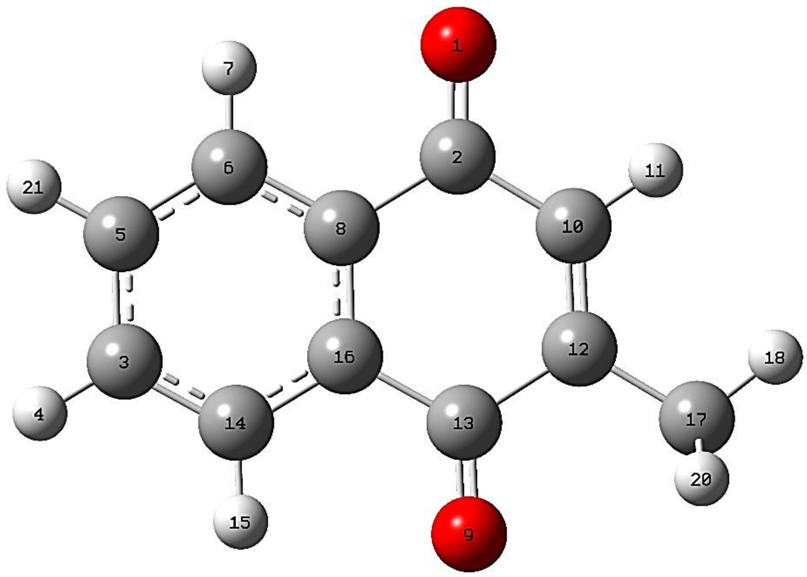


**Figure S5. The molecule of menadione optimized at B3LYP-GD3BJ/SNST level of theory.**

## Tables

**Table S1. Geometrical parameters in Cartesian coordinates of menadione optimized at B3LYP-GD3BJ/SNST level of theory.**

| | | | |
|---|---|---|---|
| 8 | -0.057309000 | 2.906484000 | 0.000000000 |
| 6 | 0.236110000 | 1.720442000 | 0.000000000 |
| 6 | -2.738445000 | -1.345110000 | 0.000000000 |
| 1 | -3.495820000 | -2.120591000 | -0.000001000 |
| 6 | -3.116834000 | -0.002793000 | 0.000000000 |
| 6 | -2.150887000 | 0.994500000 | 0.000000000 |
| 1 | -2.420855000 | 2.043329000 | 0.000001000 |
| 6 | -0.800295000 | 0.653315000 | 0.000000000 |
| 8 | 1.371841000 | -2.244913000 | 0.000001000 |
| 6 | 1.647752000 | 1.298550000 | -0.000001000 |
| 1 | 2.369265000 | 2.109487000 | -0.000001000 |
| 6 | 2.039727000 | 0.014213000 | 0.000000000 |
| 6 | 1.018811000 | -1.076845000 | 0.000000000 |
| 6 | -1.394234000 | -1.691595000 | 0.000000000 |
| 1 | -1.079082000 | -2.727628000 | -0.000001000 |
| 6 | -0.418743000 | -0.695453000 | 0.000000000 |
| 6 | 3.471963000 | -0.410630000 | 0.000000000 |
| 1 | 4.137518000 | 0.452665000 | 0.000001000 |
| 1 | 3.685460000 | -1.032147000 | -0.873605000 |
| 1 | 3.685459000 | -1.032149000 | 0.873603000 |
| 1 | -4.167759000 | 0.262894000 | 0.000000000 |

**Table S2. Potential energy distributions over the internal coordinates of menadione resulting from harmonic analysis performed at B3LYP-GD3BJ/SNST level of theory. Refer to Figure S4 for the numbering of the atomic centers.**

Normal Mode 1

| Name | Definition | Value | Relative Weight (%) |
|---|---|---|---|
| D1 | D(1,2,8,6) | 0.0974 | 4.9 |
| D2 | D(1,2,8,16) | 0.0919 | 4.6 |
| D3 | D(10,2,8,6) | 0.1108 | 5.5 |
| D4 | D(10,2,8,16) | 0.1053 | 5.3 |
| D5 | D(1,2,10,11) | -0.0691 | 3.5 |
| D6 | D(1,2,10,12) | -0.0787 | 3.9 |
| D7 | D(8,2,10,11) | -0.0823 | 4.1 |
| D8 | D(8,2,10,12) | -0.0919 | 4.6 |
| D21 | D(5,6,8,2) | -0.0157 | 0.8 |
| D29 | D(2,10,12,13) | -0.0269 | 1.3 |
| D31 | D(11,10,12,13) | -0.0372 | 1.9 |
| D32 | D(11,10,12,17) | -0.0181 | 0.9 |
| D33 | D(10,12,13,9) | 0.1261 | 6.3 |
| D34 | D(10,12,13,16) | 0.1306 | 6.5 |
| D35 | D(17,12,13,9) | 0.1084 | 5.4 |
| D36 | D(17,12,13,16) | 0.1128 | 5.7 |
| D37 | D(10,12,17,18) | -0.0155 | 0.8 |
| D43 | D(9,13,16,8) | -0.1103 | 5.5 |
| D44 | D(9,13,16,14) | -0.1168 | 5.8 |
| D45 | D(12,13,16,8) | -0.1148 | 5.7 |
| D46 | D(12,13,16,14) | -0.1213 | 6.1 |
| D47 | D(3,14,16,8) | 0.0126 | 0.6 |
| D48 | D(3,14,16,13) | 0.0191 | 1.0 |

Normal Mode 2

| Name | Definition | Value | Relative Weight (%) |
|---|---|---|---|
| D1 | D(1,2,8,6) | 0.1002 | 3.4 |
| D2 | D(1,2,8,16) | 0.138 | 4.7 |
| D3 | D(10,2,8,6) | 0.0766 | 2.6 |
| D4 | D(10,2,8,16) | 0.1144 | 3.9 |
| D5 | D(1,2,10,11) | -0.0482 | 1.6 |
| D6 | D(1,2,10,12) | -0.0401 | 1.4 |
| D7 | D(8,2,10,11) | -0.0249 | 0.8 |
| D9 | D(4,3,5,6) | -0.0386 | 1.3 |
| D11 | D(14,3,5,6) | -0.0602 | 2.0 |
| D12 | D(14,3,5,21) | -0.0394 | 1.3 |
| D13 | D(4,3,14,15) | 0.0236 | 0.8 |
| D15 | D(5,3,14,15) | 0.0452 | 1.5 |
| D16 | D(5,3,14,16) | 0.0233 | 0.8 |
| D17 | D(3,5,6,7) | 0.0428 | 1.5 |
| D18 | D(3,5,6,8) | 0.0237 | 0.8 |
| D19 | D(21,5,6,7) | 0.022 | 0.7 |
| D21 | D(5,6,8,2) | 0.0867 | 2.9 |
| D22 | D(5,6,8,16) | 0.0489 | 1.7 |
| D23 | D(7,6,8,2) | 0.0682 | 2.3 |
| D24 | D(7,6,8,16) | 0.0304 | 1.0 |
| D25 | D(2,8,16,13) | -0.1737 | 5.9 |
| D26 | D(2,8,16,14) | -0.1233 | 4.2 |
| D27 | D(6,8,16,13) | -0.1359 | 4.6 |
| D28 | D(6,8,16,14) | -0.0855 | 2.9 |
| D30 | D(2,10,12,17) | -0.0311 | 1.1 |
| D32 | D(11,10,12,17) | -0.0225 | 0.8 |
| D33 | D(10,12,13,9) | -0.0675 | 2.3 |
| D34 | D(10,12,13,16) | -0.0381 | 1.3 |

| | | | |
|---|---|---|---|
| D35 | D(17,12,13,9) | -0.0555 | 1.9 |
| D36 | D(17,12,13,16) | -0.0262 | 0.9 |
| D37 | D(10,12,17,18) | 0.0448 | 1.5 |
| D38 | D(10,12,17,19) | 0.05 | 1.7 |
| D39 | D(10,12,17,20) | 0.05 | 1.7 |
| D40 | D(13,12,17,18) | 0.0324 | 1.1 |
| D41 | D(13,12,17,19) | 0.0376 | 1.3 |
| D42 | D(13,12,17,20) | 0.0376 | 1.3 |
| D43 | D(9,13,16,8) | 0.166 | 5.6 |
| D44 | D(9,13,16,14) | 0.1157 | 3.9 |
| D45 | D(12,13,16,8) | 0.1361 | 4.6 |
| D46 | D(12,13,16,14) | 0.0859 | 2.9 |
| D47 | D(3,14,16,8) | 0.0493 | 1.7 |
| D48 | D(3,14,16,13) | 0.0991 | 3.4 |
| D49 | D(15,14,16,8) | 0.028 | 0.9 |
| D50 | D(15,14,16,13) | 0.0778 | 2.6 |

---------------------------------------------------------------------------

Normal Mode 3

| Name | Definition | Value | Relative Weight (%) |
|---|---|---|---|
| D1 | D(1,2,8,6) | 0.0651 | 2.4 |
| D2 | D(1,2,8,16) | 0.0909 | 3.3 |
| D3 | D(10,2,8,6) | 0.0264 | 1.0 |
| D4 | D(10,2,8,16) | 0.0522 | 1.9 |
| D5 | D(1,2,10,11) | -0.0626 | 2.3 |
| D6 | D(1,2,10,12) | -0.1354 | 5.0 |
| D7 | D(8,2,10,11) | -0.0245 | 0.9 |
| D8 | D(8,2,10,12) | -0.0972 | 3.6 |
| D21 | D(5,6,8,2) | 0.0297 | 1.1 |
| D23 | D(7,6,8,2) | 0.0212 | 0.8 |

| | | | |
|---|---|---|---|
| D26 | D(2,8,16,14) | -0.0279 | 1.0 |
| D27 | D(6,8,16,13) | 0.0244 | 0.9 |
| D29 | D(2,10,12,13) | 0.0846 | 3.1 |
| D30 | D(2,10,12,17) | 0.1181 | 4.3 |
| D32 | D(11,10,12,17) | 0.0409 | 1.5 |
| D33 | D(10,12,13,9) | -0.026 | 1.0 |
| D34 | D(10,12,13,16) | -0.0279 | 1.0 |
| D35 | D(17,12,13,9) | -0.0572 | 2.1 |
| D36 | D(17,12,13,16) | -0.0591 | 2.2 |
| D37 | D(10,12,17,18) | -0.2383 | 8.7 |
| D38 | D(10,12,17,19) | -0.2625 | 9.6 |
| D39 | D(10,12,17,20) | -0.2625 | 9.6 |
| D40 | D(13,12,17,18) | -0.2059 | 7.5 |
| D41 | D(13,12,17,19) | -0.2301 | 8.4 |
| D42 | D(13,12,17,20) | -0.2301 | 8.4 |
| D48 | D(3,14,16,13) | -0.0281 | 1.0 |
| D50 | D(15,14,16,13) | -0.019 | 0.7 |

-----------------------------------------------------------------------------

Normal Mode 4

| Name | Definition | Value | Relative Weight (%) |
|---|---|---|---|
| D2 | D(1,2,8,16) | 0.0757 | 2.6 |
| D3 | D(10,2,8,6) | -0.0235 | 0.8 |
| D4 | D(10,2,8,16) | 0.0349 | 1.2 |
| D5 | D(1,2,10,11) | -0.0466 | 1.6 |
| D6 | D(1,2,10,12) | -0.097 | 3.3 |
| D8 | D(8,2,10,12) | -0.0568 | 1.9 |
| D21 | D(5,6,8,2) | 0.0705 | 2.4 |
| D23 | D(7,6,8,2) | 0.0476 | 1.6 |
| D26 | D(2,8,16,14) | -0.0572 | 2.0 |

| | | | |
|---|---|---|---|
| D27 | D(6,8,16,13) | 0.0632 | 2.2 |
| D29 | D(2,10,12,13) | 0.0353 | 1.2 |
| D30 | D(2,10,12,17) | 0.0541 | 1.9 |
| D33 | D(10,12,13,9) | 0.0374 | 1.3 |
| D35 | D(17,12,13,9) | 0.0199 | 0.7 |
| D37 | D(10,12,17,18) | 0.2644 | 9.0 |
| D38 | D(10,12,17,19) | 0.2795 | 9.6 |
| D39 | D(10,12,17,20) | 0.2795 | 9.6 |
| D40 | D(13,12,17,18) | 0.2826 | 9.7 |
| D41 | D(13,12,17,19) | 0.2976 | 10.2 |
| D42 | D(13,12,17,20) | 0.2976 | 10.2 |
| D43 | D(9,13,16,8) | -0.0576 | 2.0 |
| D45 | D(12,13,16,8) | -0.0268 | 0.9 |
| D46 | D(12,13,16,14) | 0.035 | 1.2 |
| D48 | D(3,14,16,13) | -0.0748 | 2.6 |
| D50 | D(15,14,16,13) | -0.0514 | 1.8 |

---------------------------------------------------------------------------

Normal Mode 5

| Name | Definition | Value | Relative Weight (%) |
|---|---|---|---|
| D1 | D(1,2,8,6) | -0.1537 | 3.3 |
| D2 | D(1,2,8,16) | -0.0331 | 0.7 |
| D3 | D(10,2,8,6) | -0.1409 | 3.1 |
| D5 | D(1,2,10,11) | 0.0363 | 0.8 |
| D6 | D(1,2,10,12) | 0.1526 | 3.3 |
| D8 | D(8,2,10,12) | 0.1399 | 3.0 |
| D14 | D(4,3,14,16) | 0.0554 | 1.2 |
| D16 | D(5,3,14,16) | 0.0533 | 1.2 |
| D18 | D(3,5,6,8) | -0.0454 | 1.0 |
| D20 | D(21,5,6,8) | -0.0571 | 1.2 |

| | | | |
|---|---|---|---|
| D21 | D(5,6,8,2) | 0.1794 | 3.9 |
| D22 | D(5,6,8,16) | 0.0589 | 1.3 |
| D23 | D(7,6,8,2) | 0.1168 | 2.5 |
| D25 | D(2,8,16,13) | -0.0336 | 0.7 |
| D26 | D(2,8,16,14) | -0.1371 | 3.0 |
| D27 | D(6,8,16,13) | 0.087 | 1.9 |
| D29 | D(2,10,12,13) | -0.1929 | 4.2 |
| D30 | D(2,10,12,17) | -0.1688 | 3.7 |
| D31 | D(11,10,12,13) | -0.0694 | 1.5 |
| D32 | D(11,10,12,17) | -0.0454 | 1.0 |
| D33 | D(10,12,13,9) | 0.1891 | 4.1 |
| D34 | D(10,12,13,16) | 0.1282 | 2.8 |
| D35 | D(17,12,13,9) | 0.1667 | 3.6 |
| D36 | D(17,12,13,16) | 0.1058 | 2.3 |
| D37 | D(10,12,17,18) | -0.2485 | 5.4 |
| D38 | D(10,12,17,19) | -0.2483 | 5.4 |
| D39 | D(10,12,17,20) | -0.2483 | 5.4 |
| D40 | D(13,12,17,18) | -0.2252 | 4.9 |
| D41 | D(13,12,17,19) | -0.225 | 4.9 |
| D42 | D(13,12,17,20) | -0.225 | 4.9 |
| D43 | D(9,13,16,8) | -0.0773 | 1.7 |
| D46 | D(12,13,16,14) | 0.0879 | 1.9 |
| D47 | D(3,14,16,8) | -0.0396 | 0.9 |
| D48 | D(3,14,16,13) | -0.1419 | 3.1 |
| D50 | D(15,14,16,13) | -0.0893 | 1.9 |

----------------------------------------------------------------------------

Normal Mode 6

| Name | Definition | Value | Relative Weight (%) |
|---|---|---|---|
| R2 | R(2,8) | -0.0286 | 2.7 |

| | | | |
|---|---|---|---|
| R3 | R(2,10) | -0.0347 | 3.2 |
| R6 | R(3,14) | 0.0172 | 1.6 |
| R7 | R(5,6) | -0.0153 | 1.4 |
| R10 | R(6,8) | -0.0093 | 0.9 |
| R15 | R(12,13) | 0.0349 | 3.2 |
| R17 | R(13,16) | 0.0414 | 3.8 |
| R19 | R(14,16) | 0.0119 | 1.1 |
| A1 | A(1,2,8) | -0.0221 | 2.0 |
| A2 | A(1,2,10) | 0.0186 | 1.7 |
| A4 | A(4,3,5) | -0.0084 | 0.8 |
| A5 | A(4,3,14) | -0.0126 | 1.2 |
| A6 | A(5,3,14) | 0.0209 | 1.9 |
| A7 | A(3,5,6) | -0.0224 | 2.1 |
| A8 | A(3,5,21) | 0.0097 | 0.9 |
| A9 | A(6,5,21) | 0.0127 | 1.2 |
| A10 | A(5,6,7) | 0.0161 | 1.5 |
| A12 | A(7,6,8) | -0.0152 | 1.4 |
| A13 | A(2,8,6) | -0.0801 | 7.4 |
| A14 | A(2,8,16) | 0.0363 | 3.4 |
| A15 | A(6,8,16) | 0.0438 | 4.1 |
| A16 | A(2,10,11) | 0.0113 | 1.0 |
| A17 | A(2,10,12) | -0.0068 | 0.6 |
| A19 | A(10,12,13) | 0.017 | 1.6 |
| A20 | A(10,12,17) | -0.0793 | 7.3 |
| A21 | A(13,12,17) | 0.0623 | 5.8 |
| A23 | A(9,13,16) | 0.0249 | 2.3 |
| A24 | A(12,13,16) | -0.0183 | 1.7 |
| A25 | A(3,14,15) | -0.0181 | 1.7 |
| A27 | A(15,14,16) | 0.0129 | 1.2 |
| A28 | A(8,16,13) | -0.0316 | 2.9 |
| A29 | A(8,16,14) | -0.0466 | 4.3 |

| | | | |
|---|---|---|---|
| A30 | A(13,16,14) | 0.0783 | 7.2 |
| A31 | A(12,17,18) | -0.024 | 2.2 |
| A32 | A(12,17,19) | 0.0144 | 1.3 |
| A33 | A(12,17,20) | 0.0144 | 1.3 |
| A36 | A(19,17,20) | 0.0068 | 0.6 |
| D38 | D(10,12,17,19) | 0.0129 | 1.2 |
| D39 | D(10,12,17,20) | -0.0129 | 1.2 |
| D41 | D(13,12,17,19) | 0.0129 | 1.2 |
| D42 | D(13,12,17,20) | -0.0129 | 1.2 |

----------------------------------------------------------------------------

Normal Mode 7

------------------------- -------------------------

| Name | Definition | Value | Relative Weight (%) |
|---|---|---|---|
| R2 | R(2,8) | 0.01 | 0.8 |
| R3 | R(2,10) | -0.0115 | 0.9 |
| R7 | R(5,6) | 0.0114 | 0.9 |
| R10 | R(6,8) | 0.0113 | 0.9 |
| R11 | R(8,16) | 0.0095 | 0.7 |
| R14 | R(10,12) | -0.0085 | 0.7 |
| R16 | R(12,17) | 0.0082 | 0.6 |
| R17 | R(13,16) | 0.027 | 2.1 |
| A1 | A(1,2,8) | 0.0648 | 5.0 |
| A2 | A(1,2,10) | -0.059 | 4.6 |
| A6 | A(5,3,14) | -0.0089 | 0.7 |
| A10 | A(5,6,7) | -0.0109 | 0.8 |
| A13 | A(2,8,6) | 0.0654 | 5.1 |
| A14 | A(2,8,16) | -0.0493 | 3.8 |
| A15 | A(6,8,16) | -0.0162 | 1.2 |
| A16 | A(2,10,11) | -0.0384 | 3.0 |
| A17 | A(2,10,12) | 0.0609 | 4.7 |

| | | | |
|---|---|---|---|
| A18 | A(11,10,12) | -0.0225 | 1.7 |
| A19 | A(10,12,13) | -0.0398 | 3.1 |
| A20 | A(10,12,17) | -0.1033 | 8.0 |
| A21 | A(13,12,17) | 0.1432 | 11.1 |
| A22 | A(9,13,12) | 0.0574 | 4.4 |
| A23 | A(9,13,16) | -0.0396 | 3.1 |
| A24 | A(12,13,16) | -0.0178 | 1.4 |
| A26 | A(3,14,16) | 0.0099 | 0.8 |
| A27 | A(15,14,16) | -0.0105 | 0.8 |
| A28 | A(8,16,13) | 0.0518 | 4.0 |
| A30 | A(13,16,14) | -0.0582 | 4.5 |
| A31 | A(12,17,18) | -0.0383 | 3.0 |
| A32 | A(12,17,19) | 0.0228 | 1.8 |
| A33 | A(12,17,20) | 0.0228 | 1.8 |
| A34 | A(18,17,19) | -0.0098 | 0.8 |
| A35 | A(18,17,20) | -0.0098 | 0.8 |
| A36 | A(19,17,20) | 0.0143 | 1.1 |
| D38 | D(10,12,17,19) | 0.0226 | 1.7 |
| D39 | D(10,12,17,20) | -0.0226 | 1.7 |
| D41 | D(13,12,17,19) | 0.0226 | 1.7 |
| D42 | D(13,12,17,20) | -0.0226 | 1.7 |

---------------------------------------------------------------------------

Normal Mode 8

| Name | Definition | Value | Relative Weight (%) |
|---|---|---|---|
| A32 | A(12,17,19) | -0.0562 | 0.9 |
| A33 | A(12,17,20) | 0.0562 | 0.9 |
| D1 | D(1,2,8,6) | 0.0584 | 0.9 |
| D2 | D(1,2,8,16) | 0.1044 | 1.6 |
| D4 | D(10,2,8,16) | 0.0747 | 1.2 |

| | | | |
|---|---|---|---|
| D5 | D(1,2,10,11) | -0.2329 | 3.6 |
| D6 | D(1,2,10,12) | -0.2693 | 4.2 |
| D7 | D(8,2,10,11) | -0.2036 | 3.2 |
| D8 | D(8,2,10,12) | -0.24 | 3.7 |
| D9 | D(4,3,5,6) | 0.082 | 1.3 |
| D11 | D(14,3,5,6) | 0.0957 | 1.5 |
| D12 | D(14,3,5,21) | 0.043 | 0.7 |
| D14 | D(4,3,14,16) | 0.0832 | 1.3 |
| D16 | D(5,3,14,16) | 0.0695 | 1.1 |
| D17 | D(3,5,6,7) | -0.0944 | 1.5 |
| D18 | D(3,5,6,8) | -0.1571 | 2.4 |
| D19 | D(21,5,6,7) | -0.0417 | 0.6 |
| D20 | D(21,5,6,8) | -0.1044 | 1.6 |
| D21 | D(5,6,8,2) | 0.1001 | 1.6 |
| D22 | D(5,6,8,16) | 0.0542 | 0.8 |
| D26 | D(2,8,16,14) | 0.0641 | 1.0 |
| D27 | D(6,8,16,13) | 0.0712 | 1.1 |
| D28 | D(6,8,16,14) | 0.1101 | 1.7 |
| D29 | D(2,10,12,13) | 0.2855 | 4.4 |
| D30 | D(2,10,12,17) | -0.144 | 2.2 |
| D31 | D(11,10,12,13) | 0.2469 | 3.8 |
| D32 | D(11,10,12,17) | -0.1826 | 2.8 |
| D33 | D(10,12,13,9) | -0.3698 | 5.7 |
| D34 | D(10,12,13,16) | -0.1684 | 2.6 |
| D36 | D(17,12,13,16) | 0.2322 | 3.6 |
| D37 | D(10,12,17,18) | 0.2574 | 4.0 |
| D38 | D(10,12,17,19) | 0.3055 | 4.7 |
| D39 | D(10,12,17,20) | 0.3055 | 4.7 |
| D40 | D(13,12,17,18) | -0.1588 | 2.5 |
| D41 | D(13,12,17,19) | -0.1108 | 1.7 |
| D42 | D(13,12,17,20) | -0.1108 | 1.7 |

| | | | |
|---|---|---|---|
| D43 | D(9,13,16,8) | 0.2189 | 3.4 |
| D44 | D(9,13,16,14) | 0.18 | 2.8 |
| D47 | D(3,14,16,8) | -0.1716 | 2.7 |
| D48 | D(3,14,16,13) | -0.1331 | 2.1 |
| D49 | D(15,14,16,8) | -0.0985 | 1.5 |
| D50 | D(15,14,16,13) | -0.06 | 0.9 |

---------------------------------------------------------------------------

Normal Mode 9

| Name | Definition | Value | Relative Weight (%) |
|---|---|---|---|
| R2 | R(2,8) | 0.1007 | 4.7 |
| R6 | R(3,14) | 0.036 | 1.7 |
| R7 | R(5,6) | 0.0283 | 1.3 |
| R10 | R(6,8) | 0.0451 | 2.1 |
| R11 | R(8,16) | 0.0409 | 1.9 |
| R14 | R(10,12) | -0.0433 | 2.0 |
| R15 | R(12,13) | -0.0308 | 1.4 |
| R17 | R(13,16) | 0.129 | 6.0 |
| R19 | R(14,16) | 0.0422 | 2.0 |
| A1 | A(1,2,8) | 0.0959 | 4.5 |
| A2 | A(1,2,10) | -0.1737 | 8.1 |
| A3 | A(8,2,10) | 0.0778 | 3.6 |
| A10 | A(5,6,7) | -0.0337 | 1.6 |
| A11 | A(5,6,8) | 0.045 | 2.1 |
| A13 | A(2,8,6) | 0.0825 | 3.9 |
| A14 | A(2,8,16) | -0.0679 | 3.2 |
| A16 | A(2,10,11) | -0.0427 | 2.0 |
| A18 | A(11,10,12) | 0.0462 | 2.2 |
| A19 | A(10,12,13) | -0.0297 | 1.4 |
| A20 | A(10,12,17) | 0.0566 | 2.6 |

| | | | |
|---|---|---|---|
| A21 | A(13,12,17) | -0.0269 | 1.3 |
| A22 | A(9,13,12) | -0.1829 | 8.6 |
| A23 | A(9,13,16) | 0.1022 | 4.8 |
| A24 | A(12,13,16) | 0.0807 | 3.8 |
| A25 | A(3,14,15) | -0.0421 | 2.0 |
| A26 | A(3,14,16) | 0.0494 | 2.3 |
| A28 | A(8,16,13) | -0.0574 | 2.7 |
| A29 | A(8,16,14) | -0.0437 | 2.0 |
| A30 | A(13,16,14) | 0.1011 | 4.7 |

----------------------------------------------------------------------------

Normal Mode 10

------------------------- -------------------------

| Name | Definition | Value | Relative Weight (%) |
|---|---|---|---|
| R2 | R(2,8) | -0.0802 | 3.8 |
| R3 | R(2,10) | 0.1107 | 5.2 |
| R10 | R(6,8) | 0.0424 | 2.0 |
| R11 | R(8,16) | -0.0592 | 2.8 |
| R14 | R(10,12) | 0.014 | 0.7 |
| R15 | R(12,13) | 0.0252 | 1.2 |
| R16 | R(12,17) | 0.0894 | 4.2 |
| R17 | R(13,16) | 0.0736 | 3.5 |
| A1 | A(1,2,8) | -0.116 | 5.5 |
| A3 | A(8,2,10) | 0.1138 | 5.3 |
| A5 | A(4,3,14) | -0.0287 | 1.3 |
| A6 | A(5,3,14) | 0.0377 | 1.8 |
| A7 | A(3,5,6) | -0.0418 | 2.0 |
| A9 | A(6,5,21) | 0.0299 | 1.4 |
| A10 | A(5,6,7) | 0.0202 | 0.9 |
| A11 | A(5,6,8) | -0.0248 | 1.2 |
| A13 | A(2,8,6) | 0.0507 | 2.4 |

| | | | |
|---|---|---|---|
| A14 | A(2,8,16) | -0.126 | 5.9 |
| A15 | A(6,8,16) | 0.0753 | 3.5 |
| A16 | A(2,10,11) | -0.0569 | 2.7 |
| A17 | A(2,10,12) | 0.0321 | 1.5 |
| A18 | A(11,10,12) | 0.0248 | 1.2 |
| A19 | A(10,12,13) | -0.1703 | 8.0 |
| A20 | A(10,12,17) | 0.1167 | 5.5 |
| A21 | A(13,12,17) | 0.0536 | 2.5 |
| A22 | A(9,13,12) | 0.0148 | 0.7 |
| A23 | A(9,13,16) | -0.1161 | 5.5 |
| A24 | A(12,13,16) | 0.1013 | 4.8 |
| A25 | A(3,14,15) | -0.0207 | 1.0 |
| A28 | A(8,16,13) | 0.0491 | 2.3 |
| A29 | A(8,16,14) | -0.0587 | 2.8 |

----------------------------------------------------------------------------

Normal Mode 11

| Name | Definition | Value | Relative Weight (%) |
|---|---|---|---|
| D1 | D(1,2,8,6) | -0.1482 | 2.4 |
| D2 | D(1,2,8,16) | 0.1232 | 2.0 |
| D3 | D(10,2,8,6) | -0.2484 | 4.0 |
| D5 | D(1,2,10,11) | -0.1301 | 2.1 |
| D6 | D(1,2,10,12) | -0.1232 | 2.0 |
| D9 | D(4,3,5,6) | -0.047 | 0.8 |
| D11 | D(14,3,5,6) | 0.086 | 1.4 |
| D12 | D(14,3,5,21) | 0.1609 | 2.6 |
| D13 | D(4,3,14,15) | -0.1052 | 1.7 |
| D14 | D(4,3,14,16) | -0.2177 | 3.5 |
| D15 | D(5,3,14,15) | -0.2382 | 3.8 |
| D16 | D(5,3,14,16) | -0.3507 | 5.7 |

| | | | |
|---|---|---|---|
| D17 | D(3,5,6,7) | 0.1602 | 2.6 |
| D18 | D(3,5,6,8) | 0.263 | 4.2 |
| D19 | D(21,5,6,7) | 0.0853 | 1.4 |
| D20 | D(21,5,6,8) | 0.1881 | 3.0 |
| D21 | D(5,6,8,2) | -0.0747 | 1.2 |
| D22 | D(5,6,8,16) | -0.3459 | 5.6 |
| D24 | D(7,6,8,16) | -0.2461 | 4.0 |
| D26 | D(2,8,16,14) | -0.1894 | 3.1 |
| D27 | D(6,8,16,13) | 0.2357 | 3.8 |
| D28 | D(6,8,16,14) | 0.0819 | 1.3 |
| D30 | D(2,10,12,17) | -0.1202 | 1.9 |
| D31 | D(11,10,12,13) | 0.0444 | 0.7 |
| D32 | D(11,10,12,17) | -0.1129 | 1.8 |
| D33 | D(10,12,13,9) | -0.0463 | 0.7 |
| D34 | D(10,12,13,16) | -0.047 | 0.8 |
| D35 | D(17,12,13,9) | 0.1004 | 1.6 |
| D36 | D(17,12,13,16) | 0.0997 | 1.6 |
| D37 | D(10,12,17,18) | 0.045 | 0.7 |
| D38 | D(10,12,17,19) | 0.074 | 1.2 |
| D39 | D(10,12,17,20) | 0.074 | 1.2 |
| D40 | D(13,12,17,18) | -0.1075 | 1.7 |
| D41 | D(13,12,17,19) | -0.0784 | 1.3 |
| D42 | D(13,12,17,20) | -0.0784 | 1.3 |
| D43 | D(9,13,16,8) | 0.046 | 0.7 |
| D44 | D(9,13,16,14) | 0.1994 | 3.2 |
| D45 | D(12,13,16,8) | 0.0467 | 0.8 |
| D46 | D(12,13,16,14) | 0.2001 | 3.2 |
| D47 | D(3,14,16,8) | 0.2661 | 4.3 |
| D48 | D(3,14,16,13) | 0.114 | 1.8 |
| D49 | D(15,14,16,8) | 0.1566 | 2.5 |

Normal Mode 12

| Name | Definition | Value | Relative Weight (%) |
|---|---|---|---|
| R1 | R(1,2) | 0.0185 | 0.6 |
| R2 | R(2,8) | -0.0797 | 2.7 |
| R3 | R(2,10) | 0.0702 | 2.4 |
| R5 | R(3,5) | -0.0193 | 0.7 |
| R10 | R(6,8) | 0.0244 | 0.8 |
| R11 | R(8,16) | 0.0863 | 2.9 |
| R12 | R(9,13) | 0.0299 | 1.0 |
| R14 | R(10,12) | 0.0356 | 1.2 |
| R15 | R(12,13) | -0.1053 | 3.6 |
| R17 | R(13,16) | 0.1172 | 4.0 |
| R19 | R(14,16) | -0.0692 | 2.3 |
| A1 | A(1,2,8) | 0.0937 | 3.2 |
| A2 | A(1,2,10) | 0.0391 | 1.3 |
| A3 | A(8,2,10) | -0.1328 | 4.5 |
| A5 | A(4,3,14) | -0.0538 | 1.8 |
| A6 | A(5,3,14) | 0.0664 | 2.2 |
| A7 | A(3,5,6) | -0.0731 | 2.5 |
| A8 | A(3,5,21) | 0.0209 | 0.7 |
| A9 | A(6,5,21) | 0.0522 | 1.8 |
| A10 | A(5,6,7) | 0.0234 | 0.8 |
| A14 | A(2,8,16) | -0.0768 | 2.6 |
| A15 | A(6,8,16) | 0.084 | 2.8 |
| A16 | A(2,10,11) | -0.0846 | 2.9 |
| A17 | A(2,10,12) | 0.1922 | 6.5 |
| A18 | A(11,10,12) | -0.1076 | 3.6 |
| A20 | A(10,12,17) | 0.0539 | 1.8 |
| A21 | A(13,12,17) | -0.0427 | 1.4 |

| | | | |
|---|---|---|---|
| A22 | A(9,13,12) | 0.0252 | 0.9 |
| A23 | A(9,13,16) | 0.1628 | 5.5 |
| A24 | A(12,13,16) | -0.188 | 6.4 |
| A25 | A(3,14,15) | -0.0405 | 1.4 |
| A26 | A(3,14,16) | 0.0543 | 1.8 |
| A28 | A(8,16,13) | 0.2166 | 7.3 |
| A29 | A(8,16,14) | -0.1243 | 4.2 |
| A30 | A(13,16,14) | -0.0923 | 3.1 |
| A31 | A(12,17,18) | 0.0228 | 0.8 |

----------------------------------------------------------------------------

Normal Mode 13

| Name | Definition | Value | Relative Weight (%) |
|---|---|---|---|
| D2 | D(1,2,8,16) | 0.2531 | 3.2 |
| D3 | D(10,2,8,6) | -0.0956 | 1.2 |
| D4 | D(10,2,8,16) | 0.1563 | 2.0 |
| D6 | D(1,2,10,12) | 0.057 | 0.7 |
| D7 | D(8,2,10,11) | 0.106 | 1.3 |
| D8 | D(8,2,10,12) | 0.1523 | 1.9 |
| D9 | D(4,3,5,6) | 0.3305 | 4.2 |
| D10 | D(4,3,5,21) | 0.1529 | 1.9 |
| D11 | D(14,3,5,6) | 0.4535 | 5.7 |
| D12 | D(14,3,5,21) | 0.276 | 3.5 |
| D14 | D(4,3,14,16) | -0.0846 | 1.1 |
| D15 | D(5,3,14,15) | -0.1338 | 1.7 |
| D16 | D(5,3,14,16) | -0.2076 | 2.6 |
| D17 | D(3,5,6,7) | -0.2302 | 2.9 |
| D18 | D(3,5,6,8) | -0.3126 | 4.0 |
| D19 | D(21,5,6,7) | -0.0527 | 0.7 |
| D20 | D(21,5,6,8) | -0.1351 | 1.7 |

| | | | |
|---|---|---|---|
| D21 | D(5,6,8,2) | 0.1816 | 2.3 |
| D22 | D(5,6,8,16) | -0.0702 | 0.9 |
| D23 | D(7,6,8,2) | 0.1016 | 1.3 |
| D24 | D(7,6,8,16) | -0.1502 | 1.9 |
| D25 | D(2,8,16,13) | -0.3261 | 4.1 |
| D26 | D(2,8,16,14) | 0.062 | 0.8 |
| D27 | D(6,8,16,13) | -0.0742 | 0.9 |
| D28 | D(6,8,16,14) | 0.3139 | 4.0 |
| D29 | D(2,10,12,13) | -0.2698 | 3.4 |
| D31 | D(11,10,12,13) | -0.2207 | 2.8 |
| D33 | D(10,12,13,9) | 0.0804 | 1.0 |
| D34 | D(10,12,13,16) | 0.0911 | 1.2 |
| D35 | D(17,12,13,9) | -0.1662 | 2.1 |
| D36 | D(17,12,13,16) | -0.1556 | 2.0 |
| D37 | D(10,12,17,18) | -0.1302 | 1.6 |
| D38 | D(10,12,17,19) | -0.1528 | 1.9 |
| D39 | D(10,12,17,20) | -0.1528 | 1.9 |
| D40 | D(13,12,17,18) | 0.1262 | 1.6 |
| D41 | D(13,12,17,19) | 0.1035 | 1.3 |
| D42 | D(13,12,17,20) | 0.1035 | 1.3 |
| D43 | D(9,13,16,8) | 0.2205 | 2.8 |
| D44 | D(9,13,16,14) | -0.1666 | 2.1 |
| D45 | D(12,13,16,8) | 0.2097 | 2.7 |
| D46 | D(12,13,16,14) | -0.1774 | 2.2 |
| D47 | D(3,14,16,8) | -0.1745 | 2.2 |
| D48 | D(3,14,16,13) | 0.2092 | 2.6 |
| D49 | D(15,14,16,8) | -0.2464 | 3.1 |
| D50 | D(15,14,16,13) | 0.1373 | 1.7 |

---------------------------------------------------------------------------

Normal Mode 14

------------------------                    ------------------------

| Name | Definition | Value | Relative Weight (%) |
|---|---|---|---|
| R1 | R(1,2) | 0.0245 | 0.8 |
| R2 | R(2,8) | 0.1668 | 5.6 |
| R3 | R(2,10) | 0.1536 | 5.2 |
| R5 | R(3,5) | -0.0204 | 0.7 |
| R6 | R(3,14) | 0.0298 | 1.0 |
| R7 | R(5,6) | 0.035 | 1.2 |
| R10 | R(6,8) | 0.024 | 0.8 |
| R11 | R(8,16) | 0.0421 | 1.4 |
| R14 | R(10,12) | 0.085 | 2.9 |
| R15 | R(12,13) | 0.1854 | 6.3 |
| R16 | R(12,17) | 0.1075 | 3.6 |
| R17 | R(13,16) | 0.0782 | 2.6 |
| R19 | R(14,16) | 0.077 | 2.6 |
| A2 | A(1,2,10) | 0.1503 | 5.1 |
| A3 | A(8,2,10) | -0.1318 | 4.4 |
| A4 | A(4,3,5) | 0.0712 | 2.4 |
| A5 | A(4,3,14) | 0.0322 | 1.1 |
| A6 | A(5,3,14) | -0.1034 | 3.5 |
| A8 | A(3,5,21) | 0.0501 | 1.7 |
| A9 | A(6,5,21) | -0.0332 | 1.1 |
| A10 | A(5,6,7) | -0.1017 | 3.4 |
| A11 | A(5,6,8) | 0.1528 | 5.2 |
| A12 | A(7,6,8) | -0.0511 | 1.7 |
| A14 | A(2,8,16) | 0.1317 | 4.4 |
| A15 | A(6,8,16) | -0.1351 | 4.6 |
| A16 | A(2,10,11) | 0.028 | 0.9 |
| A17 | A(2,10,12) | 0.0264 | 0.9 |
| A18 | A(11,10,12) | -0.0544 | 1.8 |
| A19 | A(10,12,13) | 0.0435 | 1.5 |

| | | | |
|---|---|---|---|
| A21 | A(13,12,17) | -0.0452 | 1.5 |
| A22 | A(9,13,12) | 0.064 | 2.2 |
| A24 | A(12,13,16) | -0.047 | 1.6 |
| A25 | A(3,14,15) | -0.0642 | 2.2 |
| A26 | A(3,14,16) | 0.1222 | 4.1 |
| A27 | A(15,14,16) | -0.058 | 2.0 |
| A28 | A(8,16,13) | -0.0228 | 0.8 |
| A29 | A(8,16,14) | -0.0196 | 0.7 |
| A30 | A(13,16,14) | 0.0424 | 1.4 |
| A31 | A(12,17,18) | 0.0222 | 0.7 |

Normal Mode 15

| Name | Definition | Value | Relative Weight (%) |
|---|---|---|---|
| R3 | R(2,10) | 0.0496 | 1.5 |
| R6 | R(3,14) | -0.0263 | 0.8 |
| R7 | R(5,6) | 0.0365 | 1.1 |
| R10 | R(6,8) | 0.034 | 1.0 |
| R11 | R(8,16) | -0.0341 | 1.0 |
| R15 | R(12,13) | -0.1166 | 3.6 |
| R16 | R(12,17) | -0.0393 | 1.2 |
| R19 | R(14,16) | -0.0262 | 0.8 |
| A1 | A(1,2,8) | -0.2155 | 6.6 |
| A2 | A(1,2,10) | 0.1938 | 5.9 |
| A3 | A(8,2,10) | 0.0217 | 0.7 |
| A4 | A(4,3,5) | 0.0619 | 1.9 |
| A5 | A(4,3,14) | 0.0407 | 1.2 |
| A6 | A(5,3,14) | -0.1026 | 3.1 |
| A7 | A(3,5,6) | 0.0502 | 1.5 |
| A9 | A(6,5,21) | -0.0444 | 1.4 |

| | | | |
|---|---|---|---|
| A10 | A(5,6,7) | -0.0586 | 1.8 |
| A11 | A(5,6,8) | 0.0274 | 0.8 |
| A12 | A(7,6,8) | 0.0312 | 1.0 |
| A13 | A(2,8,6) | 0.1235 | 3.8 |
| A14 | A(2,8,16) | -0.0242 | 0.7 |
| A15 | A(6,8,16) | -0.0993 | 3.0 |
| A16 | A(2,10,11) | 0.1068 | 3.3 |
| A17 | A(2,10,12) | -0.0693 | 2.1 |
| A18 | A(11,10,12) | -0.0376 | 1.2 |
| A19 | A(10,12,13) | 0.0635 | 1.9 |
| A20 | A(10,12,17) | -0.189 | 5.8 |
| A21 | A(13,12,17) | 0.1255 | 3.9 |
| A22 | A(9,13,12) | -0.1592 | 4.9 |
| A23 | A(9,13,16) | 0.1646 | 5.1 |
| A26 | A(3,14,16) | 0.064 | 2.0 |
| A27 | A(15,14,16) | -0.073 | 2.2 |
| A29 | A(8,16,14) | 0.0603 | 1.8 |
| A30 | A(13,16,14) | -0.074 | 2.3 |
| A31 | A(12,17,18) | -0.1184 | 3.6 |
| A32 | A(12,17,19) | 0.0612 | 1.9 |
| A33 | A(12,17,20) | 0.0612 | 1.9 |
| A36 | A(19,17,20) | 0.0285 | 0.9 |
| D38 | D(10,12,17,19) | 0.0547 | 1.7 |
| D39 | D(10,12,17,20) | -0.0547 | 1.7 |
| D41 | D(13,12,17,19) | 0.0547 | 1.7 |
| D42 | D(13,12,17,20) | -0.0547 | 1.7 |

---------------------------------------------------------------------------

Normal Mode 16

------------------------- -------------------------

| Name | Definition | Value | Relative Weight (%) |
|---|---|---|---|

---------------------------------------------------------------------------

| | | | |
|---|---|---|---|
| R1 | R(1,2) | -0.0297 | 0.8 |
| R2 | R(2,8) | -0.0491 | 1.2 |
| R3 | R(2,10) | -0.0844 | 2.1 |
| R5 | R(3,5) | -0.0399 | 1.0 |
| R10 | R(6,8) | 0.0377 | 1.0 |
| R11 | R(8,16) | -0.1073 | 2.7 |
| R16 | R(12,17) | -0.1614 | 4.1 |
| R17 | R(13,16) | -0.0656 | 1.7 |
| R19 | R(14,16) | -0.058 | 1.5 |
| A2 | A(1,2,10) | -0.065 | 1.7 |
| A3 | A(8,2,10) | 0.0841 | 2.1 |
| A4 | A(4,3,5) | 0.1019 | 2.6 |
| A5 | A(4,3,14) | -0.0781 | 2.0 |
| A7 | A(3,5,6) | -0.2071 | 5.3 |
| A8 | A(3,5,21) | 0.1469 | 3.7 |
| A9 | A(6,5,21) | 0.0602 | 1.5 |
| A10 | A(5,6,7) | -0.0797 | 2.0 |
| A11 | A(5,6,8) | 0.2023 | 5.1 |
| A12 | A(7,6,8) | -0.1227 | 3.1 |
| A13 | A(2,8,6) | 0.0819 | 2.1 |
| A14 | A(2,8,16) | -0.0607 | 1.5 |
| A16 | A(2,10,11) | 0.0451 | 1.1 |
| A17 | A(2,10,12) | -0.1067 | 2.7 |
| A18 | A(11,10,12) | 0.0616 | 1.6 |
| A19 | A(10,12,13) | 0.0996 | 2.5 |
| A21 | A(13,12,17) | -0.1039 | 2.6 |
| A22 | A(9,13,12) | 0.1515 | 3.8 |
| A23 | A(9,13,16) | -0.0339 | 0.9 |
| A24 | A(12,13,16) | -0.1176 | 3.0 |
| A25 | A(3,14,15) | -0.1347 | 3.4 |
| A26 | A(3,14,16) | 0.2162 | 5.5 |

| | | | |
|---|---|---|---|
| A27 | A(15,14,16) | -0.0815 | 2.1 |
| A28 | A(8,16,13) | 0.1013 | 2.6 |
| A29 | A(8,16,14) | -0.1664 | 4.2 |
| A30 | A(13,16,14) | 0.0651 | 1.7 |
| A31 | A(12,17,18) | 0.0681 | 1.7 |
| A32 | A(12,17,19) | -0.0381 | 1.0 |
| A33 | A(12,17,20) | -0.0381 | 1.0 |
| A36 | A(19,17,20) | -0.0257 | 0.7 |
| D38 | D(10,12,17,19) | -0.0389 | 1.0 |
| D39 | D(10,12,17,20) | 0.0389 | 1.0 |
| D41 | D(13,12,17,19) | -0.0389 | 1.0 |
| D42 | D(13,12,17,20) | 0.0389 | 1.0 |

Normal Mode 17

| Name | Definition | Value | Relative Weight (%) |
|---|---|---|---|
| A32 | A(12,17,19) | 0.0801 | 1.1 |
| A33 | A(12,17,20) | -0.0801 | 1.1 |
| D1 | D(1,2,8,6) | -0.3015 | 4.1 |
| D2 | D(1,2,8,16) | -0.2642 | 3.6 |
| D3 | D(10,2,8,6) | 0.1748 | 2.4 |
| D4 | D(10,2,8,16) | 0.2122 | 2.9 |
| D5 | D(1,2,10,11) | 0.0552 | 0.8 |
| D6 | D(1,2,10,12) | 0.1931 | 2.6 |
| D7 | D(8,2,10,11) | -0.4141 | 5.6 |
| D8 | D(8,2,10,12) | -0.2762 | 3.8 |
| D11 | D(14,3,5,6) | 0.2529 | 3.4 |
| D12 | D(14,3,5,21) | 0.31 | 4.2 |
| D15 | D(5,3,14,15) | -0.3041 | 4.1 |
| D16 | D(5,3,14,16) | -0.2761 | 3.7 |

| | | | |
|---|---|---|---|
| D18 | D(3,5,6,8) | -0.2015 | 2.7 |
| D20 | D(21,5,6,8) | -0.2586 | 3.5 |
| D21 | D(5,6,8,2) | 0.2118 | 2.9 |
| D22 | D(5,6,8,16) | 0.1745 | 2.4 |
| D24 | D(7,6,8,16) | -0.0625 | 0.8 |
| D26 | D(2,8,16,14) | -0.2352 | 3.2 |
| D27 | D(6,8,16,13) | 0.0535 | 0.7 |
| D28 | D(6,8,16,14) | -0.1979 | 2.7 |
| D29 | D(2,10,12,13) | 0.0966 | 1.3 |
| D30 | D(2,10,12,17) | 0.1635 | 2.2 |
| D31 | D(11,10,12,13) | 0.2429 | 3.3 |
| D32 | D(11,10,12,17) | 0.3098 | 4.2 |
| D33 | D(10,12,13,9) | -0.0479 | 0.7 |
| D34 | D(10,12,13,16) | 0.1424 | 1.9 |
| D35 | D(17,12,13,9) | -0.1103 | 1.5 |
| D36 | D(17,12,13,16) | 0.08 | 1.1 |
| D37 | D(10,12,17,18) | 0.0514 | 0.7 |
| D40 | D(13,12,17,18) | 0.1162 | 1.6 |
| D44 | D(9,13,16,14) | 0.2473 | 3.4 |
| D45 | D(12,13,16,8) | -0.1969 | 2.7 |
| D46 | D(12,13,16,14) | 0.0538 | 0.7 |
| D47 | D(3,14,16,8) | 0.2484 | 3.4 |
| D49 | D(15,14,16,8) | 0.2757 | 3.7 |

---

Normal Mode 18

| Name | Definition | Value | Relative Weight (%) |
|---|---|---|---|
| A32 | A(12,17,19) | 0.1009 | 1.1 |
| A33 | A(12,17,20) | -0.1009 | 1.1 |
| D1 | D(1,2,8,6) | -0.2727 | 2.9 |

| | | | |
|---|---|---|---|
| D2 | D(1,2,8,16) | 0.114 | 1.2 |
| D3 | D(10,2,8,6) | -0.1573 | 1.7 |
| D4 | D(10,2,8,16) | 0.2294 | 2.5 |
| D5 | D(1,2,10,11) | -0.0744 | 0.8 |
| D6 | D(1,2,10,12) | 0.1211 | 1.3 |
| D7 | D(8,2,10,11) | -0.1881 | 2.0 |
| D9 | D(4,3,5,6) | -0.3452 | 3.7 |
| D11 | D(14,3,5,6) | -0.3504 | 3.8 |
| D14 | D(4,3,14,16) | 0.3373 | 3.6 |
| D15 | D(5,3,14,15) | 0.0631 | 0.7 |
| D16 | D(5,3,14,16) | 0.3425 | 3.7 |
| D17 | D(3,5,6,7) | 0.3725 | 4.0 |
| D18 | D(3,5,6,8) | 0.3816 | 4.1 |
| D22 | D(5,6,8,16) | -0.4061 | 4.4 |
| D24 | D(7,6,8,16) | -0.3973 | 4.3 |
| D25 | D(2,8,16,13) | -0.1792 | 1.9 |
| D27 | D(6,8,16,13) | 0.2075 | 2.2 |
| D28 | D(6,8,16,14) | 0.3986 | 4.3 |
| D29 | D(2,10,12,13) | -0.2818 | 3.0 |
| D31 | D(11,10,12,13) | -0.0743 | 0.8 |
| D32 | D(11,10,12,17) | 0.2487 | 2.7 |
| D33 | D(10,12,13,9) | -0.0783 | 0.8 |
| D34 | D(10,12,13,16) | 0.3237 | 3.5 |
| D35 | D(17,12,13,9) | -0.3795 | 4.1 |
| D38 | D(10,12,17,19) | -0.146 | 1.6 |
| D39 | D(10,12,17,20) | -0.146 | 1.6 |
| D40 | D(13,12,17,18) | 0.2595 | 2.8 |
| D41 | D(13,12,17,19) | 0.167 | 1.8 |
| D42 | D(13,12,17,20) | 0.167 | 1.8 |
| D43 | D(9,13,16,8) | 0.318 | 3.4 |
| D44 | D(9,13,16,14) | 0.1273 | 1.4 |

| | | | |
|---|---|---|---|
| D45 | D(12,13,16,8) | -0.0907 | 1.0 |
| D46 | D(12,13,16,14) | -0.2814 | 3.0 |
| D47 | D(3,14,16,8) | -0.3664 | 3.9 |
| D48 | D(3,14,16,13) | -0.1774 | 1.9 |
| D49 | D(15,14,16,8) | -0.0944 | 1.0 |
| D50 | D(15,14,16,13) | 0.0946 | 1.0 |

---------------------------------------------------------------------------

Normal Mode 19

| Name | Definition | Value | Relative Weight (%) |
|---|---|---|---|
| R2 | R(2,8) | 0.1666 | 4.0 |
| R3 | R(2,10) | 0.0415 | 1.0 |
| R5 | R(3,5) | 0.0506 | 1.2 |
| R7 | R(5,6) | 0.0328 | 0.8 |
| R10 | R(6,8) | 0.1491 | 3.5 |
| R11 | R(8,16) | 0.1161 | 2.8 |
| R12 | R(9,13) | 0.0434 | 1.0 |
| R15 | R(12,13) | 0.2106 | 5.0 |
| R16 | R(12,17) | -0.0893 | 2.1 |
| R17 | R(13,16) | 0.1109 | 2.6 |
| A1 | A(1,2,8) | -0.1677 | 4.0 |
| A3 | A(8,2,10) | 0.1815 | 4.3 |
| A4 | A(4,3,5) | -0.1081 | 2.6 |
| A5 | A(4,3,14) | -0.0416 | 1.0 |
| A6 | A(5,3,14) | 0.1497 | 3.6 |
| A7 | A(3,5,6) | 0.0372 | 0.9 |
| A8 | A(3,5,21) | -0.1032 | 2.4 |
| A9 | A(6,5,21) | 0.066 | 1.6 |
| A10 | A(5,6,7) | 0.07 | 1.7 |
| A11 | A(5,6,8) | -0.1707 | 4.1 |

| | | | |
|---|---|---|---|
| A12 | A(7,6,8) | 0.1007 | 2.4 |
| A13 | A(2,8,6) | 0.0272 | 0.6 |
| A14 | A(2,8,16) | -0.1101 | 2.6 |
| A15 | A(6,8,16) | 0.0828 | 2.0 |
| A16 | A(2,10,11) | 0.1138 | 2.7 |
| A17 | A(2,10,12) | -0.1957 | 4.6 |
| A18 | A(11,10,12) | 0.0819 | 1.9 |
| A19 | A(10,12,13) | 0.1766 | 4.2 |
| A20 | A(10,12,17) | -0.0723 | 1.7 |
| A21 | A(13,12,17) | -0.1044 | 2.5 |
| A22 | A(9,13,12) | 0.0725 | 1.7 |
| A23 | A(9,13,16) | 0.0311 | 0.7 |
| A24 | A(12,13,16) | -0.1036 | 2.5 |
| A25 | A(3,14,15) | 0.0556 | 1.3 |
| A26 | A(3,14,16) | -0.1428 | 3.4 |
| A27 | A(15,14,16) | 0.0872 | 2.1 |
| A28 | A(8,16,13) | 0.0513 | 1.2 |
| A29 | A(8,16,14) | 0.0437 | 1.0 |
| A30 | A(13,16,14) | -0.095 | 2.3 |
| A31 | A(12,17,18) | 0.0589 | 1.4 |
| D38 | D(10,12,17,19) | -0.0293 | 0.7 |
| D39 | D(10,12,17,20) | 0.0293 | 0.7 |
| D41 | D(13,12,17,19) | -0.0293 | 0.7 |
| D42 | D(13,12,17,20) | 0.0293 | 0.7 |

---

Normal Mode 20

| Name | Definition | Value | Relative Weight (%) |
|---|---|---|---|
| A32 | A(12,17,19) | -0.0443 | 0.8 |
| A33 | A(12,17,20) | 0.0443 | 0.8 |

| | | | |
|---|---|---|---|
| D1 | D(1,2,8,6) | 0.1795 | 3.2 |
| D3 | D(10,2,8,6) | 0.0413 | 0.7 |
| D4 | D(10,2,8,16) | -0.1149 | 2.0 |
| D5 | D(1,2,10,11) | 0.0788 | 1.4 |
| D6 | D(1,2,10,12) | -0.0567 | 1.0 |
| D7 | D(8,2,10,11) | 0.2149 | 3.8 |
| D8 | D(8,2,10,12) | 0.0794 | 1.4 |
| D9 | D(4,3,5,6) | -0.3807 | 6.8 |
| D12 | D(14,3,5,21) | 0.3768 | 6.7 |
| D13 | D(4,3,14,15) | 0.1587 | 2.8 |
| D14 | D(4,3,14,16) | 0.4072 | 7.2 |
| D15 | D(5,3,14,15) | -0.2408 | 4.3 |
| D17 | D(3,5,6,7) | 0.1691 | 3.0 |
| D18 | D(3,5,6,8) | -0.0634 | 1.1 |
| D19 | D(21,5,6,7) | -0.1884 | 3.3 |
| D20 | D(21,5,6,8) | -0.4209 | 7.5 |
| D21 | D(5,6,8,2) | -0.0753 | 1.3 |
| D22 | D(5,6,8,16) | 0.0808 | 1.4 |
| D23 | D(7,6,8,2) | -0.3012 | 5.4 |
| D24 | D(7,6,8,16) | -0.1452 | 2.6 |
| D25 | D(2,8,16,13) | 0.0667 | 1.2 |
| D26 | D(2,8,16,14) | 0.1021 | 1.8 |
| D27 | D(6,8,16,13) | -0.0895 | 1.6 |
| D28 | D(6,8,16,14) | -0.054 | 1.0 |
| D31 | D(11,10,12,13) | -0.1375 | 2.4 |
| D32 | D(11,10,12,17) | -0.1687 | 3.0 |
| D34 | D(10,12,13,16) | -0.0575 | 1.0 |
| D35 | D(17,12,13,9) | 0.0363 | 0.6 |
| D40 | D(13,12,17,18) | -0.0501 | 0.9 |
| D43 | D(9,13,16,8) | -0.0462 | 0.8 |
| D44 | D(9,13,16,14) | -0.0815 | 1.4 |

| | | | |
|---|---|---|---|
| D48 | D(3,14,16,13) | 0.0449 | 0.8 |
| D49 | D(15,14,16,8) | 0.2516 | 4.5 |
| D50 | D(15,14,16,13) | 0.2867 | 5.1 |

------------------------------------------------------------------------------

Normal Mode 21

| Name | Definition | Value | Relative Weight (%) |
|---|---|---|---|
| R2 | R(2,8) | -0.2231 | 5.2 |
| R3 | R(2,10) | -0.1357 | 3.2 |
| R10 | R(6,8) | -0.0602 | 1.4 |
| R12 | R(9,13) | 0.0277 | 0.6 |
| R15 | R(12,13) | 0.1685 | 3.9 |
| R17 | R(13,16) | 0.1714 | 4.0 |
| A2 | A(1,2,10) | -0.0802 | 1.9 |
| A3 | A(8,2,10) | 0.1059 | 2.5 |
| A4 | A(4,3,5) | 0.1005 | 2.3 |
| A5 | A(4,3,14) | 0.1774 | 4.1 |
| A6 | A(5,3,14) | -0.2779 | 6.5 |
| A7 | A(3,5,6) | 0.2615 | 6.1 |
| A8 | A(3,5,21) | -0.08 | 1.9 |
| A9 | A(6,5,21) | -0.1815 | 4.2 |
| A10 | A(5,6,7) | -0.0348 | 0.8 |
| A11 | A(5,6,8) | -0.0899 | 2.1 |
| A12 | A(7,6,8) | 0.1246 | 2.9 |
| A13 | A(2,8,6) | 0.0678 | 1.6 |
| A15 | A(6,8,16) | -0.0501 | 1.2 |
| A16 | A(2,10,11) | -0.0984 | 2.3 |
| A18 | A(11,10,12) | 0.0798 | 1.9 |
| A20 | A(10,12,17) | 0.1026 | 2.4 |
| A21 | A(13,12,17) | -0.0798 | 1.9 |

| | | | |
|---|---|---|---|
| A22 | A(9,13,12) | 0.0419 | 1.0 |
| A23 | A(9,13,16) | 0.0763 | 1.8 |
| A24 | A(12,13,16) | -0.1182 | 2.8 |
| A25 | A(3,14,15) | 0.0283 | 0.7 |
| A26 | A(3,14,16) | 0.1178 | 2.8 |
| A27 | A(15,14,16) | -0.1461 | 3.4 |
| A28 | A(8,16,13) | 0.0342 | 0.8 |
| A29 | A(8,16,14) | 0.0386 | 0.9 |
| A30 | A(13,16,14) | -0.0728 | 1.7 |
| A31 | A(12,17,18) | 0.1358 | 3.2 |
| A32 | A(12,17,19) | -0.0695 | 1.6 |
| A33 | A(12,17,20) | -0.0695 | 1.6 |
| A36 | A(19,17,20) | -0.0392 | 0.9 |
| D38 | D(10,12,17,19) | -0.0663 | 1.5 |
| D39 | D(10,12,17,20) | 0.0663 | 1.5 |
| D41 | D(13,12,17,19) | -0.0663 | 1.5 |
| D42 | D(13,12,17,20) | 0.0663 | 1.5 |

---------------------------------------------------------------------------

Normal Mode 22

| Name | Definition | Value | Relative Weight (%) |
|---|---|---|---|
| A32 | A(12,17,19) | 0.0779 | 0.7 |
| A33 | A(12,17,20) | -0.0779 | 0.7 |
| D1 | D(1,2,8,6) | 0.3738 | 3.3 |
| D4 | D(10,2,8,16) | -0.4588 | 4.1 |
| D5 | D(1,2,10,11) | -0.1373 | 1.2 |
| D7 | D(8,2,10,11) | 0.2631 | 2.3 |
| D8 | D(8,2,10,12) | 0.3788 | 3.4 |
| D10 | D(4,3,5,21) | -0.2649 | 2.4 |
| D11 | D(14,3,5,6) | 0.112 | 1.0 |

| | | | |
|---|---|---|---|
| D12 | D(14,3,5,21) | -0.1684 | 1.5 |
| D13 | D(4,3,14,15) | -0.2131 | 1.9 |
| D14 | D(4,3,14,16) | -0.0919 | 0.8 |
| D15 | D(5,3,14,15) | -0.3094 | 2.7 |
| D16 | D(5,3,14,16) | -0.1883 | 1.7 |
| D17 | D(3,5,6,7) | -0.3783 | 3.4 |
| D18 | D(3,5,6,8) | -0.165 | 1.5 |
| D19 | D(21,5,6,7) | -0.098 | 0.9 |
| D20 | D(21,5,6,8) | 0.1153 | 1.0 |
| D21 | D(5,6,8,2) | -0.1319 | 1.2 |
| D22 | D(5,6,8,16) | 0.294 | 2.6 |
| D23 | D(7,6,8,2) | 0.0753 | 0.7 |
| D24 | D(7,6,8,16) | 0.5012 | 4.4 |
| D25 | D(2,8,16,13) | 0.5786 | 5.1 |
| D27 | D(6,8,16,13) | 0.1525 | 1.4 |
| D28 | D(6,8,16,14) | -0.37 | 3.3 |
| D29 | D(2,10,12,13) | -0.4008 | 3.6 |
| D30 | D(2,10,12,17) | -0.0879 | 0.8 |
| D31 | D(11,10,12,13) | -0.278 | 2.5 |
| D33 | D(10,12,13,9) | -0.0953 | 0.8 |
| D34 | D(10,12,13,16) | 0.4841 | 4.3 |
| D35 | D(17,12,13,9) | -0.3871 | 3.4 |
| D36 | D(17,12,13,16) | 0.1923 | 1.7 |
| D38 | D(10,12,17,19) | -0.1365 | 1.2 |
| D39 | D(10,12,17,20) | -0.1365 | 1.2 |
| D40 | D(13,12,17,18) | 0.235 | 2.1 |
| D41 | D(13,12,17,19) | 0.1667 | 1.5 |
| D42 | D(13,12,17,20) | 0.1667 | 1.5 |
| D44 | D(9,13,16,14) | 0.5288 | 4.7 |
| D45 | D(12,13,16,8) | -0.5814 | 5.2 |
| D47 | D(3,14,16,8) | 0.3167 | 2.8 |

| | | | |
|---|---|---|---|
| D48 | D(3,14,16,13) | -0.2 | 1.8 |
| D49 | D(15,14,16,8) | 0.4345 | 3.9 |
| D50 | D(15,14,16,13) | -0.0821 | 0.7 |

---------------------------------------------------------------------------

Normal Mode 23

------------------------- -------------------------

| Name | Definition | Value | Relative Weight (%) |
|---|---|---|---|
| A32 | A(12,17,19) | -0.0586 | 0.8 |
| A33 | A(12,17,20) | 0.0586 | 0.8 |
| D2 | D(1,2,8,16) | -0.0625 | 0.8 |
| D3 | D(10,2,8,6) | 0.095 | 1.3 |
| D5 | D(1,2,10,11) | 0.4169 | 5.7 |
| D7 | D(8,2,10,11) | 0.3566 | 4.8 |
| D8 | D(8,2,10,12) | -0.0752 | 1.0 |
| D9 | D(4,3,5,6) | 0.2576 | 3.5 |
| D10 | D(4,3,5,21) | 0.433 | 5.9 |
| D12 | D(14,3,5,21) | 0.1632 | 2.2 |
| D13 | D(4,3,14,15) | 0.0621 | 0.8 |
| D14 | D(4,3,14,16) | -0.3021 | 4.1 |
| D15 | D(5,3,14,15) | 0.3317 | 4.5 |
| D17 | D(3,5,6,7) | 0.3603 | 4.9 |
| D19 | D(21,5,6,7) | 0.185 | 2.5 |
| D20 | D(21,5,6,8) | -0.2035 | 2.8 |
| D22 | D(5,6,8,16) | 0.1128 | 1.5 |
| D23 | D(7,6,8,2) | -0.3609 | 4.9 |
| D24 | D(7,6,8,16) | -0.2647 | 3.6 |
| D25 | D(2,8,16,13) | 0.1116 | 1.5 |
| D26 | D(2,8,16,14) | -0.0608 | 0.8 |
| D28 | D(6,8,16,14) | -0.1571 | 2.1 |
| D30 | D(2,10,12,17) | 0.0654 | 0.9 |

| | | | |
|---|---|---|---|
| D31 | D(11,10,12,13) | -0.4233 | 5.7 |
| D32 | D(11,10,12,17) | -0.3929 | 5.3 |
| D33 | D(10,12,13,9) | -0.0815 | 1.1 |
| D34 | D(10,12,13,16) | 0.0765 | 1.0 |
| D35 | D(17,12,13,9) | -0.1098 | 1.5 |
| D36 | D(17,12,13,16) | 0.0482 | 0.7 |
| D41 | D(13,12,17,19) | 0.0706 | 1.0 |
| D42 | D(13,12,17,20) | 0.0706 | 1.0 |
| D44 | D(9,13,16,14) | 0.1821 | 2.5 |
| D45 | D(12,13,16,8) | -0.1504 | 2.0 |
| D47 | D(3,14,16,8) | 0.1168 | 1.6 |
| D48 | D(3,14,16,13) | -0.0536 | 0.7 |
| D49 | D(15,14,16,8) | -0.2377 | 3.2 |
| D50 | D(15,14,16,13) | -0.4081 | 5.5 |

---------------------------------------------------------------------------

Normal Mode 24

| Name | Definition | Value | Relative Weight (%) |
|---|---|---|---|
| A32 | A(12,17,19) | -0.1098 | 1.4 |
| A33 | A(12,17,20) | 0.1098 | 1.4 |
| D1 | D(1,2,8,6) | -0.2124 | 2.8 |
| D4 | D(10,2,8,16) | 0.2188 | 2.9 |
| D5 | D(1,2,10,11) | 0.6141 | 8.1 |
| D7 | D(8,2,10,11) | 0.3633 | 4.8 |
| D8 | D(8,2,10,12) | -0.2455 | 3.2 |
| D9 | D(4,3,5,6) | -0.1057 | 1.4 |
| D10 | D(4,3,5,21) | -0.3452 | 4.5 |
| D12 | D(14,3,5,21) | -0.2319 | 3.0 |
| D13 | D(4,3,14,15) | -0.1763 | 2.3 |
| D14 | D(4,3,14,16) | 0.1411 | 1.9 |

| | | | |
|---|---|---|---|
| D15 | D(5,3,14,15) | -0.2895 | 3.8 |
| D17 | D(3,5,6,7) | -0.2503 | 3.3 |
| D20 | D(21,5,6,8) | 0.2701 | 3.5 |
| D21 | D(5,6,8,2) | 0.0728 | 1.0 |
| D22 | D(5,6,8,16) | -0.1038 | 1.4 |
| D23 | D(7,6,8,2) | 0.3457 | 4.5 |
| D24 | D(7,6,8,16) | 0.1691 | 2.2 |
| D25 | D(2,8,16,13) | -0.121 | 1.6 |
| D27 | D(6,8,16,13) | 0.0557 | 0.7 |
| D28 | D(6,8,16,14) | 0.1389 | 1.8 |
| D29 | D(2,10,12,13) | 0.1611 | 2.1 |
| D30 | D(2,10,12,17) | 0.1286 | 1.7 |
| D31 | D(11,10,12,13) | -0.4851 | 6.4 |
| D32 | D(11,10,12,17) | -0.5175 | 6.8 |
| D33 | D(10,12,13,9) | -0.0743 | 1.0 |
| D34 | D(10,12,13,16) | -0.0483 | 0.6 |
| D38 | D(10,12,17,19) | 0.0877 | 1.2 |
| D39 | D(10,12,17,20) | 0.0877 | 1.2 |
| D40 | D(13,12,17,18) | -0.0638 | 0.8 |
| D41 | D(13,12,17,19) | 0.0563 | 0.7 |
| D42 | D(13,12,17,20) | 0.0563 | 0.7 |
| D43 | D(9,13,16,8) | 0.0577 | 0.8 |
| D46 | D(12,13,16,14) | -0.0518 | 0.7 |
| D47 | D(3,14,16,8) | -0.1008 | 1.3 |
| D49 | D(15,14,16,8) | 0.2081 | 2.7 |
| D50 | D(15,14,16,13) | 0.2904 | 3.8 |

---------------------------------------------------------------------------

Normal Mode 25

| Name | Definition | Value | Relative Weight (%) |
|---|---|---|---|

| | | | |
|---|---|---|---|
| R3 | R(2,10) | 0.0774 | 1.7 |
| R5 | R(3,5) | 0.0778 | 1.7 |
| R6 | R(3,14) | 0.1054 | 2.3 |
| R10 | R(6,8) | 0.0599 | 1.3 |
| R11 | R(8,16) | 0.0683 | 1.5 |
| R14 | R(10,12) | -0.0852 | 1.8 |
| R15 | R(12,13) | -0.2648 | 5.7 |
| R16 | R(12,17) | -0.3781 | 8.1 |
| R17 | R(13,16) | 0.1492 | 3.2 |
| R19 | R(14,16) | 0.1933 | 4.1 |
| A2 | A(1,2,10) | 0.0967 | 2.1 |
| A3 | A(8,2,10) | -0.0727 | 1.6 |
| A5 | A(4,3,14) | 0.1008 | 2.2 |
| A6 | A(5,3,14) | -0.0875 | 1.9 |
| A7 | A(3,5,6) | 0.1192 | 2.6 |
| A8 | A(3,5,21) | -0.0342 | 0.7 |
| A9 | A(6,5,21) | -0.0851 | 1.8 |
| A11 | A(5,6,8) | -0.0724 | 1.6 |
| A12 | A(7,6,8) | 0.0691 | 1.5 |
| A14 | A(2,8,16) | -0.0491 | 1.1 |
| A15 | A(6,8,16) | 0.0764 | 1.6 |
| A16 | A(2,10,11) | 0.0535 | 1.1 |
| A17 | A(2,10,12) | 0.1349 | 2.9 |
| A18 | A(11,10,12) | -0.1885 | 4.0 |
| A19 | A(10,12,13) | -0.0608 | 1.3 |
| A20 | A(10,12,17) | 0.0346 | 0.7 |
| A22 | A(9,13,12) | 0.1817 | 3.9 |
| A23 | A(9,13,16) | -0.2469 | 5.3 |
| A24 | A(12,13,16) | 0.0652 | 1.4 |
| A25 | A(3,14,15) | -0.0448 | 1.0 |
| A26 | A(3,14,16) | 0.0321 | 0.7 |

| | | | |
|---|---|---|---|
| A29 | A(8,16,14) | -0.0678 | 1.5 |
| A30 | A(13,16,14) | 0.0852 | 1.8 |
| A31 | A(12,17,18) | 0.1368 | 2.9 |
| A32 | A(12,17,19) | -0.1028 | 2.2 |
| A33 | A(12,17,20) | -0.1028 | 2.2 |
| A34 | A(18,17,19) | 0.0592 | 1.3 |
| A35 | A(18,17,20) | 0.0592 | 1.3 |
| A36 | A(19,17,20) | -0.0562 | 1.2 |
| D38 | D(10,12,17,19) | -0.097 | 2.1 |
| D39 | D(10,12,17,20) | 0.097 | 2.1 |
| D41 | D(13,12,17,19) | -0.097 | 2.1 |
| D42 | D(13,12,17,20) | 0.097 | 2.1 |

---

Normal Mode 26

| Name | Definition | Value | Relative Weight (%) |
|---|---|---|---|
| D1 | D(1,2,8,6) | 0.0964 | 1.5 |
| D3 | D(10,2,8,6) | 0.0461 | 0.7 |
| D5 | D(1,2,10,11) | -0.0733 | 1.1 |
| D8 | D(8,2,10,12) | 0.0439 | 0.7 |
| D9 | D(4,3,5,6) | 0.3245 | 4.9 |
| D12 | D(14,3,5,21) | -0.3164 | 4.8 |
| D13 | D(4,3,14,15) | -0.7267 | 11.0 |
| D14 | D(4,3,14,16) | -0.1885 | 2.9 |
| D15 | D(5,3,14,15) | -0.3999 | 6.1 |
| D16 | D(5,3,14,16) | 0.1383 | 2.1 |
| D17 | D(3,5,6,7) | 0.3994 | 6.1 |
| D18 | D(3,5,6,8) | -0.1348 | 2.0 |
| D19 | D(21,5,6,7) | 0.7132 | 10.8 |
| D20 | D(21,5,6,8) | 0.1791 | 2.7 |

| | | | |
|---|---|---|---|
| D21 | D(5,6,8,2) | 0.0533 | 0.8 |
| D22 | D(5,6,8,16) | 0.1358 | 2.1 |
| D23 | D(7,6,8,2) | -0.4656 | 7.1 |
| D24 | D(7,6,8,16) | -0.383 | 5.8 |
| D26 | D(2,8,16,14) | 0.0821 | 1.2 |
| D27 | D(6,8,16,13) | -0.0812 | 1.2 |
| D31 | D(11,10,12,13) | 0.0578 | 0.9 |
| D32 | D(11,10,12,17) | 0.0556 | 0.8 |
| D44 | D(9,13,16,14) | -0.0931 | 1.4 |
| D46 | D(12,13,16,14) | -0.0517 | 0.8 |
| D47 | D(3,14,16,8) | -0.1365 | 2.1 |
| D48 | D(3,14,16,13) | -0.0566 | 0.9 |
| D49 | D(15,14,16,8) | 0.3873 | 5.9 |
| D50 | D(15,14,16,13) | 0.4672 | 7.1 |

---------------------------------------------------------------------------

Normal Mode 27

| Name | Definition | Value | Relative Weight (%) |
|---|---|---|---|
| D9 | D(4,3,5,6) | 0.3116 | 4.9 |
| D10 | D(4,3,5,21) | 0.8711 | 13.7 |
| D11 | D(14,3,5,6) | -0.2416 | 3.8 |
| D12 | D(14,3,5,21) | 0.318 | 5.0 |
| D13 | D(4,3,14,15) | -0.7091 | 11.1 |
| D14 | D(4,3,14,16) | -0.358 | 5.6 |
| D15 | D(5,3,14,15) | -0.1563 | 2.5 |
| D16 | D(5,3,14,16) | 0.1948 | 3.1 |
| D17 | D(3,5,6,7) | -0.1649 | 2.6 |
| D18 | D(3,5,6,8) | 0.1974 | 3.1 |
| D19 | D(21,5,6,7) | -0.7243 | 11.4 |
| D20 | D(21,5,6,8) | -0.3619 | 5.7 |

| | | | |
|---|---|---|---|
| D22 | D(5,6,8,16) | -0.108 | 1.7 |
| D23 | D(7,6,8,2) | 0.2823 | 4.4 |
| D24 | D(7,6,8,16) | 0.244 | 3.8 |
| D47 | D(3,14,16,8) | -0.1051 | 1.7 |
| D49 | D(15,14,16,8) | 0.2365 | 3.7 |
| D50 | D(15,14,16,13) | 0.2726 | 4.3 |

------------------------------------------------------------------------------

Normal Mode 28

| Name | Definition | Value | Relative Weight (%) |
|---|---|---|---|
| R3 | R(2,10) | -0.08 | 2.0 |
| R14 | R(10,12) | -0.0618 | 1.6 |
| R15 | R(12,13) | 0.0813 | 2.1 |
| A1 | A(1,2,8) | -0.0537 | 1.4 |
| A2 | A(1,2,10) | 0.088 | 2.2 |
| A3 | A(8,2,10) | -0.0342 | 0.9 |
| A4 | A(4,3,5) | -0.0296 | 0.8 |
| A5 | A(4,3,14) | -0.0454 | 1.2 |
| A6 | A(5,3,14) | 0.075 | 1.9 |
| A7 | A(3,5,6) | -0.0726 | 1.8 |
| A8 | A(3,5,21) | 0.0248 | 0.6 |
| A9 | A(6,5,21) | 0.0478 | 1.2 |
| A10 | A(5,6,7) | -0.0381 | 1.0 |
| A11 | A(5,6,8) | 0.0686 | 1.7 |
| A12 | A(7,6,8) | -0.0305 | 0.8 |
| A13 | A(2,8,6) | 0.0508 | 1.3 |
| A14 | A(2,8,16) | 0.0263 | 0.7 |
| A15 | A(6,8,16) | -0.0771 | 2.0 |
| A17 | A(2,10,12) | 0.0809 | 2.1 |
| A18 | A(11,10,12) | -0.102 | 2.6 |

| | | | |
|---|---|---|---|
| A19 | A(10,12,13) | -0.0534 | 1.4 |
| A21 | A(13,12,17) | 0.0764 | 1.9 |
| A22 | A(9,13,12) | -0.0684 | 1.7 |
| A23 | A(9,13,16) | 0.0626 | 1.6 |
| A25 | A(3,14,15) | 0.0334 | 0.8 |
| A26 | A(3,14,16) | -0.0698 | 1.8 |
| A27 | A(15,14,16) | 0.0364 | 0.9 |
| A28 | A(8,16,13) | -0.0254 | 0.6 |
| A29 | A(8,16,14) | 0.0759 | 1.9 |
| A30 | A(13,16,14) | -0.0505 | 1.3 |
| A31 | A(12,17,18) | 0.4102 | 10.4 |
| A32 | A(12,17,19) | -0.2142 | 5.4 |
| A33 | A(12,17,20) | -0.2142 | 5.4 |
| A34 | A(18,17,19) | 0.0738 | 1.9 |
| A35 | A(18,17,20) | 0.0738 | 1.9 |
| A36 | A(19,17,20) | -0.1521 | 3.9 |
| D38 | D(10,12,17,19) | -0.2236 | 5.7 |
| D39 | D(10,12,17,20) | 0.2236 | 5.7 |
| D41 | D(13,12,17,19) | -0.2236 | 5.7 |
| D42 | D(13,12,17,20) | 0.2236 | 5.7 |

--------------------------------------------------------------------------------

Normal Mode 29

| Name | Definition | Value | Relative Weight (%) |
|---|---|---|---|
| R2 | R(2,8) | 0.0267 | 1.1 |
| R3 | R(2,10) | 0.0802 | 3.2 |
| R4 | R(3,4) | -0.0158 | 0.6 |
| R5 | R(3,5) | -0.2701 | 10.7 |
| R6 | R(3,14) | -0.164 | 6.5 |
| R7 | R(5,6) | -0.1811 | 7.2 |

| | | | |
|---|---|---|---|
| R8 | R(5,21) | -0.0166 | 0.7 |
| R10 | R(6,8) | -0.0188 | 0.7 |
| R11 | R(8,16) | 0.0219 | 0.9 |
| R16 | R(12,17) | -0.0648 | 2.6 |
| R17 | R(13,16) | 0.0257 | 1.0 |
| A2 | A(1,2,10) | -0.018 | 0.7 |
| A4 | A(4,3,5) | -0.0932 | 3.7 |
| A5 | A(4,3,14) | 0.062 | 2.5 |
| A6 | A(5,3,14) | 0.0311 | 1.2 |
| A7 | A(3,5,6) | 0.078 | 3.1 |
| A8 | A(3,5,21) | -0.1014 | 4.0 |
| A9 | A(6,5,21) | 0.0234 | 0.9 |
| A10 | A(5,6,7) | -0.1705 | 6.8 |
| A11 | A(5,6,8) | -0.0288 | 1.1 |
| A12 | A(7,6,8) | 0.1993 | 7.9 |
| A13 | A(2,8,6) | 0.0361 | 1.4 |
| A14 | A(2,8,16) | -0.017 | 0.7 |
| A15 | A(6,8,16) | -0.0191 | 0.8 |
| A16 | A(2,10,11) | 0.0237 | 0.9 |
| A23 | A(9,13,16) | -0.0283 | 1.1 |
| A24 | A(12,13,16) | 0.0189 | 0.7 |
| A25 | A(3,14,15) | -0.1916 | 7.6 |
| A27 | A(15,14,16) | 0.1813 | 7.2 |
| A29 | A(8,16,14) | -0.0715 | 2.8 |
| A30 | A(13,16,14) | 0.0701 | 2.8 |

---------------------------------------------------------------------------

Normal Mode 30

------------------------                    ------------------------

| Name | Definition | Value | Relative Weight (%) |
|---|---|---|---|
| A32 | A(12,17,19) | -0.39 | 7.7 |

| | | | |
|---|---|---|---|
| A33 | A(12,17,20) | 0.39 | 7.7 |
| A34 | A(18,17,19) | -0.1343 | 2.7 |
| A35 | A(18,17,20) | 0.1343 | 2.7 |
| D5 | D(1,2,10,11) | -0.2455 | 4.9 |
| D6 | D(1,2,10,12) | 0.1015 | 2.0 |
| D7 | D(8,2,10,11) | -0.2175 | 4.3 |
| D8 | D(8,2,10,12) | 0.1296 | 2.6 |
| D29 | D(2,10,12,13) | -0.2062 | 4.1 |
| D30 | D(2,10,12,17) | 0.1003 | 2.0 |
| D31 | D(11,10,12,13) | 0.1621 | 3.2 |
| D32 | D(11,10,12,17) | 0.4686 | 9.3 |
| D33 | D(10,12,13,9) | 0.0772 | 1.5 |
| D34 | D(10,12,13,16) | 0.1855 | 3.7 |
| D35 | D(17,12,13,9) | -0.2087 | 4.1 |
| D36 | D(17,12,13,16) | -0.1003 | 2.0 |
| D37 | D(10,12,17,18) | -0.4657 | 9.2 |
| D40 | D(13,12,17,18) | -0.1687 | 3.3 |
| D41 | D(13,12,17,19) | 0.2668 | 5.3 |
| D42 | D(13,12,17,20) | 0.2668 | 5.3 |
| D45 | D(12,13,16,8) | -0.0953 | 1.9 |
| D46 | D(12,13,16,14) | -0.0647 | 1.3 |

------------------------------------------------------------------------------

Normal Mode 31

| Name | Definition | Value | Relative Weight (%) |
|---|---|---|---|
| R2 | R(2,8) | 0.1529 | 3.3 |
| R3 | R(2,10) | 0.1403 | 3.0 |
| R6 | R(3,14) | -0.1135 | 2.4 |
| R7 | R(5,6) | 0.0994 | 2.1 |
| R11 | R(8,16) | -0.0625 | 1.3 |

| | | | |
|---|---|---|---|
| R17 | R(13,16) | -0.0887 | 1.9 |
| R19 | R(14,16) | -0.1337 | 2.9 |
| A1 | A(1,2,8) | 0.0751 | 1.6 |
| A2 | A(1,2,10) | -0.0459 | 1.0 |
| A3 | A(8,2,10) | -0.0292 | 0.6 |
| A4 | A(4,3,5) | 0.2002 | 4.3 |
| A5 | A(4,3,14) | -0.0856 | 1.8 |
| A6 | A(5,3,14) | -0.1146 | 2.5 |
| A7 | A(3,5,6) | 0.0876 | 1.9 |
| A8 | A(3,5,21) | -0.1659 | 3.6 |
| A9 | A(6,5,21) | 0.0783 | 1.7 |
| A10 | A(5,6,7) | 0.3121 | 6.7 |
| A11 | A(5,6,8) | -0.1887 | 4.1 |
| A12 | A(7,6,8) | -0.1234 | 2.7 |
| A13 | A(2,8,6) | -0.164 | 3.5 |
| A15 | A(6,8,16) | 0.1787 | 3.8 |
| A16 | A(2,10,11) | 0.149 | 3.2 |
| A17 | A(2,10,12) | -0.0461 | 1.0 |
| A18 | A(11,10,12) | -0.1029 | 2.2 |
| A20 | A(10,12,17) | -0.0641 | 1.4 |
| A21 | A(13,12,17) | 0.0372 | 0.8 |
| A22 | A(9,13,12) | -0.0333 | 0.7 |
| A25 | A(3,14,15) | -0.1202 | 2.6 |
| A26 | A(3,14,16) | 0.1792 | 3.8 |
| A27 | A(15,14,16) | -0.059 | 1.3 |
| A28 | A(8,16,13) | 0.0349 | 0.7 |
| A29 | A(8,16,14) | -0.1421 | 3.1 |
| A30 | A(13,16,14) | 0.1072 | 2.3 |
| A31 | A(12,17,18) | 0.1487 | 3.2 |
| A32 | A(12,17,19) | -0.0696 | 1.5 |
| A33 | A(12,17,20) | -0.0696 | 1.5 |

| | | | |
|---|---|---|---|
| A36 | A(19,17,20) | -0.0599 | 1.3 |
| D38 | D(10,12,17,19) | -0.0791 | 1.7 |
| D39 | D(10,12,17,20) | 0.0791 | 1.7 |
| D41 | D(13,12,17,19) | -0.0791 | 1.7 |
| D42 | D(13,12,17,20) | 0.0791 | 1.7 |

------------------------------------------------------------------------------

Normal Mode 32

| Name | Definition | Value | Relative Weight (%) |
|---|---|---|---|
| R2 | R(2,8) | 0.0345 | 1.0 |
| R3 | R(2,10) | 0.2715 | 7.7 |
| R6 | R(3,14) | 0.102 | 2.9 |
| R7 | R(5,6) | -0.1438 | 4.1 |
| R10 | R(6,8) | -0.1838 | 5.2 |
| R11 | R(8,16) | -0.1011 | 2.9 |
| R15 | R(12,13) | 0.0615 | 1.7 |
| R16 | R(12,17) | -0.134 | 3.8 |
| R17 | R(13,16) | 0.1078 | 3.1 |
| R19 | R(14,16) | -0.0801 | 2.3 |
| A1 | A(1,2,8) | 0.0824 | 2.3 |
| A2 | A(1,2,10) | -0.1064 | 3.0 |
| A3 | A(8,2,10) | 0.024 | 0.7 |
| A4 | A(4,3,5) | -0.1266 | 3.6 |
| A5 | A(4,3,14) | 0.1542 | 4.4 |
| A6 | A(5,3,14) | -0.0276 | 0.8 |
| A8 | A(3,5,21) | 0.1568 | 4.4 |
| A9 | A(6,5,21) | -0.1481 | 4.2 |
| A10 | A(5,6,7) | 0.0297 | 0.8 |
| A11 | A(5,6,8) | 0.0466 | 1.3 |
| A12 | A(7,6,8) | -0.0763 | 2.2 |

| | | | |
|---|---|---|---|
| A16 | A(2,10,11) | 0.0641 | 1.8 |
| A17 | A(2,10,12) | -0.0621 | 1.8 |
| A20 | A(10,12,17) | -0.0325 | 0.9 |
| A21 | A(13,12,17) | 0.0278 | 0.8 |
| A22 | A(9,13,12) | -0.0354 | 1.0 |
| A23 | A(9,13,16) | 0.0295 | 0.8 |
| A25 | A(3,14,15) | 0.2986 | 8.5 |
| A26 | A(3,14,16) | -0.0675 | 1.9 |
| A27 | A(15,14,16) | -0.231 | 6.5 |
| A28 | A(8,16,13) | 0.0438 | 1.2 |
| A29 | A(8,16,14) | 0.0374 | 1.1 |
| A30 | A(13,16,14) | -0.0812 | 2.3 |
| A31 | A(12,17,18) | 0.027 | 0.8 |
| A32 | A(12,17,19) | -0.0222 | 0.6 |
| A33 | A(12,17,20) | -0.0222 | 0.6 |

Normal Mode 33

| Name | Definition | Value | Relative Weight (%) |
|---|---|---|---|
| R2 | R(2,8) | 0.0258 | 0.8 |
| R5 | R(3,5) | 0.1011 | 3.1 |
| R7 | R(5,6) | -0.1118 | 3.4 |
| R10 | R(6,8) | 0.0402 | 1.2 |
| R15 | R(12,13) | -0.0684 | 2.1 |
| R16 | R(12,17) | 0.154 | 4.7 |
| R17 | R(13,16) | -0.084 | 2.5 |
| R19 | R(14,16) | 0.0212 | 0.6 |
| A2 | A(1,2,10) | -0.0226 | 0.7 |
| A4 | A(4,3,5) | 0.1648 | 5.0 |
| A5 | A(4,3,14) | -0.1179 | 3.6 |

| | | | |
|---|---|---|---|
| A6 | A(5,3,14) | -0.0469 | 1.4 |
| A7 | A(3,5,6) | 0.033 | 1.0 |
| A8 | A(3,5,21) | 0.2741 | 8.3 |
| A9 | A(6,5,21) | -0.3071 | 9.3 |
| A10 | A(5,6,7) | -0.2104 | 6.4 |
| A12 | A(7,6,8) | 0.2258 | 6.8 |
| A14 | A(2,8,16) | -0.0288 | 0.9 |
| A15 | A(6,8,16) | 0.0436 | 1.3 |
| A16 | A(2,10,11) | 0.1684 | 5.1 |
| A17 | A(2,10,12) | -0.0572 | 1.7 |
| A18 | A(11,10,12) | -0.1112 | 3.4 |
| A19 | A(10,12,13) | 0.0704 | 2.1 |
| A20 | A(10,12,17) | -0.0613 | 1.9 |
| A22 | A(9,13,12) | 0.024 | 0.7 |
| A24 | A(12,13,16) | -0.0237 | 0.7 |
| A25 | A(3,14,15) | -0.0792 | 2.4 |
| A26 | A(3,14,16) | 0.042 | 1.3 |
| A27 | A(15,14,16) | 0.0371 | 1.1 |
| A28 | A(8,16,13) | 0.0324 | 1.0 |
| A29 | A(8,16,14) | -0.0563 | 1.7 |
| A30 | A(13,16,14) | 0.0239 | 0.7 |
| A31 | A(12,17,18) | 0.0872 | 2.6 |
| A36 | A(19,17,20) | -0.0318 | 1.0 |
| D38 | D(10,12,17,19) | -0.0299 | 0.9 |
| D39 | D(10,12,17,20) | 0.0299 | 0.9 |
| D41 | D(13,12,17,19) | -0.0299 | 0.9 |
| D42 | D(13,12,17,20) | 0.0299 | 0.9 |

-----------------------------------------------------------------------------

Normal Mode 34

------------------------- -------------------------

Name Definition Value Relative Weight (%)

--------------------------------------------------------------------------------

| | | | |
|---|---|---|---|
| R1 | R(1,2) | 0.0229 | 0.7 |
| R2 | R(2,8) | -0.0369 | 1.1 |
| R3 | R(2,10) | 0.0651 | 1.9 |
| R5 | R(3,5) | 0.095 | 2.8 |
| R6 | R(3,14) | -0.133 | 3.9 |
| R10 | R(6,8) | -0.0385 | 1.1 |
| R15 | R(12,13) | 0.1141 | 3.3 |
| R16 | R(12,17) | -0.2075 | 6.0 |
| R17 | R(13,16) | 0.0802 | 2.3 |
| A4 | A(4,3,5) | 0.2871 | 8.4 |
| A5 | A(4,3,14) | -0.3158 | 9.2 |
| A6 | A(5,3,14) | 0.0287 | 0.8 |
| A7 | A(3,5,6) | -0.0505 | 1.5 |
| A8 | A(3,5,21) | 0.1756 | 5.1 |
| A9 | A(6,5,21) | -0.1251 | 3.6 |
| A11 | A(5,6,8) | 0.0376 | 1.1 |
| A12 | A(7,6,8) | -0.034 | 1.0 |
| A14 | A(2,8,16) | 0.0311 | 0.9 |
| A15 | A(6,8,16) | -0.0456 | 1.3 |
| A16 | A(2,10,11) | -0.1973 | 5.8 |
| A17 | A(2,10,12) | 0.0507 | 1.5 |
| A18 | A(11,10,12) | 0.1467 | 4.3 |
| A19 | A(10,12,13) | -0.0861 | 2.5 |
| A20 | A(10,12,17) | 0.0612 | 1.8 |
| A21 | A(13,12,17) | 0.0249 | 0.7 |
| A22 | A(9,13,12) | -0.0549 | 1.6 |
| A24 | A(12,13,16) | 0.0351 | 1.0 |
| A25 | A(3,14,15) | -0.1515 | 4.4 |
| A26 | A(3,14,16) | -0.0222 | 0.6 |
| A27 | A(15,14,16) | 0.1738 | 5.1 |

| | | | |
|---|---|---|---|
| A28 | A(8,16,13) | -0.0268 | 0.8 |
| A29 | A(8,16,14) | 0.0521 | 1.5 |
| A30 | A(13,16,14) | -0.0254 | 0.7 |
| A31 | A(12,17,18) | -0.0845 | 2.5 |
| A34 | A(18,17,19) | 0.0216 | 0.6 |
| A35 | A(18,17,20) | 0.0216 | 0.6 |
| A36 | A(19,17,20) | 0.0284 | 0.8 |
| D38 | D(10,12,17,19) | 0.0231 | 0.7 |
| D39 | D(10,12,17,20) | -0.0231 | 0.7 |
| D41 | D(13,12,17,19) | 0.0231 | 0.7 |
| D42 | D(13,12,17,20) | -0.0231 | 0.7 |

---------------------------------------------------------------------------

Normal Mode 35

| Name | Definition | Value | Relative Weight (%) |
|---|---|---|---|
| R1 | R(1,2) | -0.0534 | 1.3 |
| R2 | R(2,8) | -0.0785 | 1.9 |
| R6 | R(3,14) | -0.0609 | 1.4 |
| R10 | R(6,8) | -0.1268 | 3.0 |
| R11 | R(8,16) | -0.0392 | 0.9 |
| R12 | R(9,13) | 0.0307 | 0.7 |
| R15 | R(12,13) | -0.1419 | 3.4 |
| R16 | R(12,17) | 0.1379 | 3.3 |
| R17 | R(13,16) | 0.2193 | 5.2 |
| R19 | R(14,16) | 0.0971 | 2.3 |
| A2 | A(1,2,10) | -0.0523 | 1.2 |
| A3 | A(8,2,10) | 0.0465 | 1.1 |
| A5 | A(4,3,14) | -0.0684 | 1.6 |
| A6 | A(5,3,14) | 0.0498 | 1.2 |
| A7 | A(3,5,6) | -0.0494 | 1.2 |

| | | | |
|---|---|---|---|
| A9 | A(6,5,21) | 0.0342 | 0.8 |
| A10 | A(5,6,7) | 0.2118 | 5.0 |
| A11 | A(5,6,8) | 0.0749 | 1.8 |
| A12 | A(7,6,8) | -0.2867 | 6.8 |
| A13 | A(2,8,6) | 0.0684 | 1.6 |
| A15 | A(6,8,16) | -0.0554 | 1.3 |
| A16 | A(2,10,11) | 0.3633 | 8.6 |
| A17 | A(2,10,12) | -0.0546 | 1.3 |
| A18 | A(11,10,12) | -0.3087 | 7.3 |
| A19 | A(10,12,13) | 0.091 | 2.2 |
| A21 | A(13,12,17) | -0.0724 | 1.7 |
| A22 | A(9,13,12) | 0.1208 | 2.9 |
| A23 | A(9,13,16) | -0.0485 | 1.1 |
| A24 | A(12,13,16) | -0.0723 | 1.7 |
| A25 | A(3,14,15) | -0.151 | 3.6 |
| A26 | A(3,14,16) | -0.0892 | 2.1 |
| A27 | A(15,14,16) | 0.2402 | 5.7 |
| A29 | A(8,16,14) | 0.0693 | 1.6 |
| A30 | A(13,16,14) | -0.0718 | 1.7 |
| A32 | A(12,17,19) | 0.0365 | 0.9 |
| A33 | A(12,17,20) | 0.0365 | 0.9 |
| A34 | A(18,17,19) | -0.05 | 1.2 |
| A35 | A(18,17,20) | -0.05 | 1.2 |
| D38 | D(10,12,17,19) | 0.0362 | 0.9 |
| D39 | D(10,12,17,20) | -0.0362 | 0.9 |
| D41 | D(13,12,17,19) | 0.0362 | 0.9 |
| D42 | D(13,12,17,20) | -0.0362 | 0.9 |

---------------------------------------------------------------------------

Normal Mode 36

------------------------ ------------------------

| Name | Definition | Value | Relative Weight (%) |
|---|---|---|---|

-------------------------------------------------------------------------------

| | | | |
|---|---|---|---|
| R2 | R(2,8) | -0.1473 | 2.8 |
| R3 | R(2,10) | 0.2132 | 4.1 |
| R6 | R(3,14) | 0.137 | 2.6 |
| R7 | R(5,6) | 0.046 | 0.9 |
| R10 | R(6,8) | -0.081 | 1.5 |
| R11 | R(8,16) | 0.1122 | 2.1 |
| R15 | R(12,13) | 0.0492 | 0.9 |
| R17 | R(13,16) | -0.2951 | 5.6 |
| R19 | R(14,16) | 0.1517 | 2.9 |
| A1 | A(1,2,8) | 0.0366 | 0.7 |
| A2 | A(1,2,10) | -0.0891 | 1.7 |
| A3 | A(8,2,10) | 0.0525 | 1.0 |
| A4 | A(4,3,5) | -0.1355 | 2.6 |
| A5 | A(4,3,14) | 0.1594 | 3.0 |
| A8 | A(3,5,21) | 0.0654 | 1.2 |
| A9 | A(6,5,21) | -0.085 | 1.6 |
| A10 | A(5,6,7) | 0.1489 | 2.8 |
| A12 | A(7,6,8) | -0.1797 | 3.4 |
| A13 | A(2,8,6) | 0.0513 | 1.0 |
| A14 | A(2,8,16) | -0.0963 | 1.8 |
| A15 | A(6,8,16) | 0.045 | 0.9 |
| A16 | A(2,10,11) | -0.188 | 3.6 |
| A17 | A(2,10,12) | -0.0985 | 1.9 |
| A18 | A(11,10,12) | 0.2865 | 5.4 |
| A20 | A(10,12,17) | -0.1043 | 2.0 |
| A21 | A(13,12,17) | 0.0808 | 1.5 |
| A22 | A(9,13,12) | -0.1078 | 2.0 |
| A23 | A(9,13,16) | 0.1282 | 2.4 |
| A25 | A(3,14,15) | -0.3729 | 7.1 |
| A27 | A(15,14,16) | 0.3631 | 6.9 |

| | | | |
|---|---|---|---|
| A28 | A(8,16,13) | 0.1393 | 2.6 |
| A29 | A(8,16,14) | -0.0814 | 1.5 |
| A30 | A(13,16,14) | -0.0579 | 1.1 |
| A31 | A(12,17,18) | 0.108 | 2.1 |
| A32 | A(12,17,19) | -0.0429 | 0.8 |
| A33 | A(12,17,20) | -0.0429 | 0.8 |
| A36 | A(19,17,20) | -0.0884 | 1.7 |
| D38 | D(10,12,17,19) | -0.0803 | 1.5 |
| D39 | D(10,12,17,20) | 0.0803 | 1.5 |
| D41 | D(13,12,17,19) | -0.0803 | 1.5 |
| D42 | D(13,12,17,20) | 0.0803 | 1.5 |

----------------------------------------------------------------------------

Normal Mode 37

| Name | Definition | Value | Relative Weight (%) |
|---|---|---|---|
| R2 | R(2,8) | 0.4477 | 8.8 |
| R3 | R(2,10) | -0.3292 | 6.4 |
| R5 | R(3,5) | 0.0555 | 1.1 |
| R6 | R(3,14) | -0.073 | 1.4 |
| R7 | R(5,6) | -0.2033 | 4.0 |
| R10 | R(6,8) | -0.0346 | 0.7 |
| R11 | R(8,16) | -0.1543 | 3.0 |
| R15 | R(12,13) | -0.0752 | 1.5 |
| R16 | R(12,17) | 0.0457 | 0.9 |
| R17 | R(13,16) | 0.0349 | 0.7 |
| R19 | R(14,16) | 0.0772 | 1.5 |
| A1 | A(1,2,8) | -0.2043 | 4.0 |
| A2 | A(1,2,10) | 0.202 | 4.0 |
| A4 | A(4,3,5) | -0.1562 | 3.1 |
| A5 | A(4,3,14) | 0.1981 | 3.9 |

| | | | |
|---|---|---|---|
| A6 | A(5,3,14) | -0.0419 | 0.8 |
| A7 | A(3,5,6) | 0.052 | 1.0 |
| A8 | A(3,5,21) | 0.1606 | 3.1 |
| A9 | A(6,5,21) | -0.2126 | 4.2 |
| A10 | A(5,6,7) | 0.3811 | 7.5 |
| A11 | A(5,6,8) | -0.0543 | 1.1 |
| A12 | A(7,6,8) | -0.3268 | 6.4 |
| A13 | A(2,8,6) | 0.0449 | 0.9 |
| A14 | A(2,8,16) | -0.1595 | 3.1 |
| A15 | A(6,8,16) | 0.1146 | 2.2 |
| A16 | A(2,10,11) | -0.2498 | 4.9 |
| A17 | A(2,10,12) | 0.0843 | 1.6 |
| A18 | A(11,10,12) | 0.1655 | 3.2 |
| A24 | A(12,13,16) | -0.0368 | 0.7 |
| A25 | A(3,14,15) | -0.0967 | 1.9 |
| A27 | A(15,14,16) | 0.0846 | 1.7 |
| A28 | A(8,16,13) | 0.1198 | 2.3 |
| A29 | A(8,16,14) | -0.0826 | 1.6 |
| A30 | A(13,16,14) | -0.0372 | 0.7 |

---------------------------------------------------------------------------

Normal Mode 38

| Name | Definition | Value | Relative Weight (%) |
|---|---|---|---|
| R2 | R(2,8) | -0.2019 | 2.5 |
| R3 | R(2,10) | 0.2071 | 2.6 |
| R5 | R(3,5) | 0.4653 | 5.9 |
| R6 | R(3,14) | -0.4464 | 5.6 |
| R7 | R(5,6) | -0.4072 | 5.1 |
| R10 | R(6,8) | 0.4764 | 6.0 |
| R11 | R(8,16) | -0.4727 | 6.0 |

| | | | |
|---|---|---|---|
| R15 | R(12,13) | -0.1005 | 1.3 |
| R19 | R(14,16) | 0.4817 | 6.1 |
| A1 | A(1,2,8) | 0.2134 | 2.7 |
| A2 | A(1,2,10) | -0.1223 | 1.5 |
| A3 | A(8,2,10) | -0.0911 | 1.2 |
| A4 | A(4,3,5) | -0.2317 | 2.9 |
| A5 | A(4,3,14) | 0.2244 | 2.8 |
| A8 | A(3,5,21) | -0.398 | 5.0 |
| A9 | A(6,5,21) | 0.4247 | 5.4 |
| A10 | A(5,6,7) | 0.0757 | 1.0 |
| A12 | A(7,6,8) | -0.1078 | 1.4 |
| A13 | A(2,8,6) | -0.1275 | 1.6 |
| A14 | A(2,8,16) | 0.163 | 2.1 |
| A16 | A(2,10,11) | -0.0969 | 1.2 |
| A17 | A(2,10,12) | -0.1135 | 1.4 |
| A18 | A(11,10,12) | 0.2104 | 2.7 |
| A19 | A(10,12,13) | 0.0651 | 0.8 |
| A20 | A(10,12,17) | -0.1144 | 1.4 |
| A23 | A(9,13,16) | 0.1077 | 1.4 |
| A24 | A(12,13,16) | -0.0951 | 1.2 |
| A25 | A(3,14,15) | 0.2509 | 3.2 |
| A27 | A(15,14,16) | -0.2439 | 3.1 |
| A28 | A(8,16,13) | 0.0716 | 0.9 |
| A30 | A(13,16,14) | -0.1014 | 1.3 |
| A31 | A(12,17,18) | 0.1372 | 1.7 |
| A36 | A(19,17,20) | -0.1411 | 1.8 |
| D38 | D(10,12,17,19) | -0.0826 | 1.0 |
| D39 | D(10,12,17,20) | 0.0826 | 1.0 |
| D41 | D(13,12,17,19) | -0.0826 | 1.0 |
| D42 | D(13,12,17,20) | 0.0826 | 1.0 |

--------------------------------------------------------------------------------

Normal Mode 39

| Name | Definition | Value | Relative Weight (%) |
|---|---|---|---|
| R3 | R(2,10) | -0.0998 | 1.7 |
| R5 | R(3,5) | 0.0885 | 1.5 |
| R7 | R(5,6) | -0.0653 | 1.1 |
| R10 | R(6,8) | 0.101 | 1.8 |
| R11 | R(8,16) | -0.0536 | 0.9 |
| R14 | R(10,12) | -0.0898 | 1.6 |
| R15 | R(12,13) | 0.417 | 7.3 |
| R16 | R(12,17) | -0.1405 | 2.5 |
| R17 | R(13,16) | -0.2343 | 4.1 |
| R19 | R(14,16) | 0.0826 | 1.4 |
| A3 | A(8,2,10) | -0.0407 | 0.7 |
| A4 | A(4,3,5) | -0.1686 | 2.9 |
| A5 | A(4,3,14) | 0.191 | 3.3 |
| A9 | A(6,5,21) | -0.0396 | 0.7 |
| A10 | A(5,6,7) | 0.0765 | 1.3 |
| A12 | A(7,6,8) | -0.064 | 1.1 |
| A16 | A(2,10,11) | 0.3523 | 6.2 |
| A17 | A(2,10,12) | 0.16 | 2.8 |
| A18 | A(11,10,12) | -0.5123 | 8.9 |
| A19 | A(10,12,13) | -0.1789 | 3.1 |
| A20 | A(10,12,17) | 0.2314 | 4.0 |
| A21 | A(13,12,17) | -0.0525 | 0.9 |
| A22 | A(9,13,12) | -0.1348 | 2.4 |
| A23 | A(9,13,16) | 0.1523 | 2.7 |
| A25 | A(3,14,15) | -0.1043 | 1.8 |
| A27 | A(15,14,16) | 0.0698 | 1.2 |
| A28 | A(8,16,13) | 0.0887 | 1.6 |

| | | | |
|---|---|---|---|
| A29 | A(8,16,14) | -0.0403 | 0.7 |
| A30 | A(13,16,14) | -0.0484 | 0.8 |
| A31 | A(12,17,18) | -0.2374 | 4.1 |
| A36 | A(19,17,20) | 0.3075 | 5.4 |
| D38 | D(10,12,17,19) | 0.1804 | 3.2 |
| D39 | D(10,12,17,20) | -0.1804 | 3.2 |
| D41 | D(13,12,17,19) | 0.1804 | 3.2 |
| D42 | D(13,12,17,20) | -0.1804 | 3.2 |

---------------------------------------------------------------------------

Normal Mode 40

| Name | Definition | Value | Relative Weight (%) |
|---|---|---|---|
| R3 | R(2,10) | -0.029 | 0.9 |
| R15 | R(12,13) | 0.083 | 2.6 |
| R16 | R(12,17) | -0.1955 | 6.2 |
| R17 | R(13,16) | -0.0218 | 0.7 |
| A5 | A(4,3,14) | 0.0208 | 0.7 |
| A8 | A(3,5,21) | 0.0212 | 0.7 |
| A9 | A(6,5,21) | -0.0233 | 0.7 |
| A16 | A(2,10,11) | 0.03 | 1.0 |
| A17 | A(2,10,12) | 0.0406 | 1.3 |
| A18 | A(11,10,12) | -0.0706 | 2.2 |
| A19 | A(10,12,13) | -0.0423 | 1.3 |
| A20 | A(10,12,17) | 0.0476 | 1.5 |
| A22 | A(9,13,12) | -0.022 | 0.7 |
| A31 | A(12,17,18) | 0.2787 | 8.8 |
| A32 | A(12,17,19) | 0.3679 | 11.7 |
| A33 | A(12,17,20) | 0.3679 | 11.7 |
| A34 | A(18,17,19) | -0.3128 | 9.9 |
| A35 | A(18,17,20) | -0.3128 | 9.9 |

| | | | |
|---|---|---|---|
| A36 | A(19,17,20) | -0.4328 | 13.7 |
| D38 | D(10,12,17,19) | -0.0421 | 1.3 |
| D39 | D(10,12,17,20) | 0.0421 | 1.3 |
| D41 | D(13,12,17,19) | -0.0421 | 1.3 |
| D42 | D(13,12,17,20) | 0.0421 | 1.3 |

---------------------------------------------------------------------------

Normal Mode 41

| Name | Definition | Value | Relative Weight (%) |
|---|---|---|---|
| A32 | A(12,17,19) | -0.1547 | 4.9 |
| A33 | A(12,17,20) | 0.1547 | 4.9 |
| A34 | A(18,17,19) | 0.554 | 17.5 |
| A35 | A(18,17,20) | -0.554 | 17.5 |
| D37 | D(10,12,17,18) | 0.4022 | 12.7 |
| D38 | D(10,12,17,19) | -0.1936 | 6.1 |
| D39 | D(10,12,17,20) | -0.1936 | 6.1 |
| D40 | D(13,12,17,18) | 0.4079 | 12.9 |
| D41 | D(13,12,17,19) | -0.188 | 5.9 |
| D42 | D(13,12,17,20) | -0.188 | 5.9 |

---------------------------------------------------------------------------

Normal Mode 42

| Name | Definition | Value | Relative Weight (%) |
|---|---|---|---|
| R3 | R(2,10) | 0.0365 | 1.0 |
| R14 | R(10,12) | -0.0514 | 1.4 |
| A4 | A(4,3,5) | 0.0275 | 0.8 |
| A5 | A(4,3,14) | -0.0273 | 0.7 |
| A16 | A(2,10,11) | -0.0936 | 2.6 |
| A18 | A(11,10,12) | 0.1015 | 2.8 |

| | | | |
|---|---|---|---|
| A19 | A(10,12,13) | 0.0271 | 0.7 |
| A21 | A(13,12,17) | -0.0243 | 0.7 |
| A25 | A(3,14,15) | 0.0233 | 0.6 |
| A31 | A(12,17,18) | 0.2307 | 6.3 |
| A32 | A(12,17,19) | -0.0322 | 0.9 |
| A33 | A(12,17,20) | -0.0322 | 0.9 |
| A34 | A(18,17,19) | -0.366 | 10.0 |
| A35 | A(18,17,20) | -0.366 | 10.0 |
| A36 | A(19,17,20) | 0.5692 | 15.6 |
| D38 | D(10,12,17,19) | 0.3299 | 9.0 |
| D39 | D(10,12,17,20) | -0.3299 | 9.0 |
| D41 | D(13,12,17,19) | 0.3299 | 9.0 |
| D42 | D(13,12,17,20) | -0.3299 | 9.0 |

---------------------------------------------------------------------------

Normal Mode 43

| Name | Definition | Value | Relative Weight (%) |
|---|---|---|---|
| R1 | R(1,2) | 0.0425 | 0.8 |
| R2 | R(2,8) | -0.1583 | 3.1 |
| R3 | R(2,10) | 0.0589 | 1.1 |
| R6 | R(3,14) | -0.1793 | 3.5 |
| R7 | R(5,6) | 0.1821 | 3.5 |
| R10 | R(6,8) | 0.2192 | 4.3 |
| R12 | R(9,13) | -0.0364 | 0.7 |
| R15 | R(12,13) | -0.0656 | 1.3 |
| R17 | R(13,16) | 0.1615 | 3.1 |
| R19 | R(14,16) | -0.2214 | 4.3 |
| A1 | A(1,2,8) | 0.0723 | 1.4 |
| A2 | A(1,2,10) | -0.0469 | 0.9 |
| A4 | A(4,3,5) | -0.4845 | 9.4 |

| | | | |
|---|---|---|---|
| A5 | A(4,3,14) | 0.4397 | 8.6 |
| A6 | A(5,3,14) | 0.0448 | 0.9 |
| A7 | A(3,5,6) | -0.0491 | 1.0 |
| A8 | A(3,5,21) | 0.4709 | 9.2 |
| A9 | A(6,5,21) | -0.4219 | 8.2 |
| A10 | A(5,6,7) | 0.1281 | 2.5 |
| A11 | A(5,6,8) | -0.0603 | 1.2 |
| A12 | A(7,6,8) | -0.0679 | 1.3 |
| A13 | A(2,8,6) | -0.0584 | 1.1 |
| A14 | A(2,8,16) | 0.1517 | 3.0 |
| A15 | A(6,8,16) | -0.0933 | 1.8 |
| A17 | A(2,10,12) | -0.0362 | 0.7 |
| A18 | A(11,10,12) | 0.0422 | 0.8 |
| A19 | A(10,12,13) | 0.0344 | 0.7 |
| A22 | A(9,13,12) | 0.0452 | 0.9 |
| A23 | A(9,13,16) | -0.0708 | 1.4 |
| A25 | A(3,14,15) | -0.1519 | 3.0 |
| A26 | A(3,14,16) | 0.0603 | 1.2 |
| A27 | A(15,14,16) | 0.0916 | 1.8 |
| A28 | A(8,16,13) | -0.1502 | 2.9 |
| A29 | A(8,16,14) | 0.0976 | 1.9 |
| A30 | A(13,16,14) | 0.0526 | 1.0 |
| A36 | A(19,17,20) | 0.0438 | 0.9 |

----------------------------------------------------------------------------

Normal Mode 44

------------------------- -------------------------

| Name | Definition | Value | Relative Weight (%) |
|---|---|---|---|
| R2 | R(2,8) | -0.179 | 3.3 |
| R3 | R(2,10) | 0.0369 | 0.7 |
| R5 | R(3,5) | -0.1791 | 3.3 |

| | | | |
|---|---|---|---|
| R6 | R(3,14) | -0.1488 | 2.8 |
| R7 | R(5,6) | -0.1325 | 2.5 |
| R10 | R(6,8) | 0.1474 | 2.7 |
| R11 | R(8,16) | 0.3166 | 5.9 |
| R14 | R(10,12) | -0.0373 | 0.7 |
| R17 | R(13,16) | -0.1693 | 3.1 |
| R19 | R(14,16) | 0.1365 | 2.5 |
| A4 | A(4,3,5) | 0.1488 | 2.8 |
| A5 | A(4,3,14) | -0.2219 | 4.1 |
| A6 | A(5,3,14) | 0.0731 | 1.4 |
| A7 | A(3,5,6) | 0.0677 | 1.3 |
| A8 | A(3,5,21) | 0.1801 | 3.3 |
| A9 | A(6,5,21) | -0.2478 | 4.6 |
| A10 | A(5,6,7) | 0.437 | 8.1 |
| A12 | A(7,6,8) | -0.4654 | 8.6 |
| A13 | A(2,8,6) | 0.1276 | 2.4 |
| A15 | A(6,8,16) | -0.1033 | 1.9 |
| A25 | A(3,14,15) | 0.4346 | 8.1 |
| A27 | A(15,14,16) | -0.4666 | 8.7 |
| A28 | A(8,16,13) | -0.035 | 0.7 |
| A29 | A(8,16,14) | -0.0978 | 1.8 |
| A30 | A(13,16,14) | 0.1329 | 2.5 |
| A31 | A(12,17,18) | -0.0337 | 0.6 |
| A34 | A(18,17,19) | 0.04 | 0.7 |
| A35 | A(18,17,20) | 0.04 | 0.7 |
| A36 | A(19,17,20) | -0.0535 | 1.0 |

---------------------------------------------------------------------------

Normal Mode 45

------------------------- -------------------------

Name Definition Value Relative Weight (%)

---------------------------------------------------------------------------

| | | | |
|---|---|---|---|
| R1 | R(1,2) | -0.1125 | 1.3 |
| R3 | R(2,10) | 0.0674 | 0.8 |
| R5 | R(3,5) | 0.642 | 7.6 |
| R6 | R(3,14) | -0.2733 | 3.2 |
| R7 | R(5,6) | -0.3091 | 3.7 |
| R10 | R(6,8) | -0.3184 | 3.8 |
| R11 | R(8,16) | 0.6106 | 7.2 |
| R12 | R(9,13) | -0.1082 | 1.3 |
| R14 | R(10,12) | -0.169 | 2.0 |
| R16 | R(12,17) | 0.0701 | 0.8 |
| R19 | R(14,16) | -0.348 | 4.1 |
| A1 | A(1,2,8) | -0.1112 | 1.3 |
| A2 | A(1,2,10) | 0.0589 | 0.7 |
| A4 | A(4,3,5) | -0.3561 | 4.2 |
| A5 | A(4,3,14) | 0.5116 | 6.1 |
| A6 | A(5,3,14) | -0.1555 | 1.8 |
| A7 | A(3,5,6) | -0.1402 | 1.7 |
| A8 | A(3,5,21) | -0.3845 | 4.6 |
| A9 | A(6,5,21) | 0.5247 | 6.2 |
| A10 | A(5,6,7) | -0.1751 | 2.1 |
| A11 | A(5,6,8) | 0.2805 | 3.3 |
| A12 | A(7,6,8) | -0.1054 | 1.3 |
| A13 | A(2,8,6) | 0.2302 | 2.7 |
| A14 | A(2,8,16) | -0.0903 | 1.1 |
| A15 | A(6,8,16) | -0.1398 | 1.7 |
| A16 | A(2,10,11) | -0.1219 | 1.4 |
| A18 | A(11,10,12) | 0.0892 | 1.1 |
| A19 | A(10,12,13) | 0.0614 | 0.7 |
| A21 | A(13,12,17) | -0.075 | 0.9 |
| A22 | A(9,13,12) | 0.0702 | 0.8 |
| A23 | A(9,13,16) | -0.1201 | 1.4 |

| | | | |
|---|---|---|---|
| A25 | A(3,14,15) | -0.2055 | 2.4 |
| A26 | A(3,14,16) | 0.2779 | 3.3 |
| A27 | A(15,14,16) | -0.0725 | 0.9 |
| A28 | A(8,16,13) | -0.106 | 1.3 |
| A29 | A(8,16,14) | -0.1229 | 1.5 |
| A30 | A(13,16,14) | 0.2289 | 2.7 |
| A31 | A(12,17,18) | -0.0582 | 0.7 |

------------------------------------------------------------------------------

Normal Mode 46

| Name | Definition | Value | Relative Weight (%) |
|---|---|---|---|
| R1 | R(1,2) | 0.1617 | 2.1 |
| R2 | R(2,8) | -0.2429 | 3.1 |
| R6 | R(3,14) | 0.5253 | 6.7 |
| R7 | R(5,6) | -0.5106 | 6.5 |
| R10 | R(6,8) | 0.4836 | 6.2 |
| R12 | R(9,13) | -0.1527 | 2.0 |
| R17 | R(13,16) | 0.235 | 3.0 |
| R19 | R(14,16) | -0.4568 | 5.8 |
| A2 | A(1,2,10) | -0.054 | 0.7 |
| A4 | A(4,3,5) | 0.3844 | 4.9 |
| A5 | A(4,3,14) | -0.1668 | 2.1 |
| A6 | A(5,3,14) | -0.2175 | 2.8 |
| A7 | A(3,5,6) | 0.2273 | 2.9 |
| A8 | A(3,5,21) | -0.3625 | 4.6 |
| A9 | A(6,5,21) | 0.1352 | 1.7 |
| A10 | A(5,6,7) | 0.4451 | 5.7 |
| A12 | A(7,6,8) | -0.4694 | 6.0 |
| A13 | A(2,8,6) | 0.0676 | 0.9 |
| A14 | A(2,8,16) | 0.1845 | 2.4 |

| | | | |
|---|---|---|---|
| A15 | A(6,8,16) | -0.2521 | 3.2 |
| A16 | A(2,10,11) | 0.0583 | 0.7 |
| A22 | A(9,13,12) | 0.0527 | 0.7 |
| A25 | A(3,14,15) | -0.4291 | 5.5 |
| A27 | A(15,14,16) | 0.469 | 6.0 |
| A28 | A(8,16,13) | -0.1787 | 2.3 |
| A29 | A(8,16,14) | 0.2579 | 3.3 |
| A30 | A(13,16,14) | -0.0792 | 1.0 |

Normal Mode 47

| Name | Definition | Value | Relative Weight (%) |
|---|---|---|---|
| R1 | R(1,2) | 0.3145 | 4.9 |
| R2 | R(2,8) | -0.0706 | 1.1 |
| R3 | R(2,10) | 0.2562 | 4.0 |
| R5 | R(3,5) | -0.0627 | 1.0 |
| R6 | R(3,14) | 0.0355 | 0.5 |
| R7 | R(5,6) | 0.0568 | 0.9 |
| R11 | R(8,16) | -0.0891 | 1.4 |
| R12 | R(9,13) | 0.3015 | 4.7 |
| R13 | R(10,11) | -0.0575 | 0.9 |
| R14 | R(10,12) | -1.0294 | 15.9 |
| R15 | R(12,13) | 0.1426 | 2.2 |
| R16 | R(12,17) | 0.4046 | 6.3 |
| R17 | R(13,16) | -0.0594 | 0.9 |
| R19 | R(14,16) | 0.0384 | 0.6 |
| A1 | A(1,2,8) | -0.0349 | 0.5 |
| A2 | A(1,2,10) | 0.0838 | 1.3 |
| A3 | A(8,2,10) | -0.0489 | 0.8 |
| A4 | A(4,3,5) | 0.0232 | 0.4 |

| | | | |
|---|---|---|---|
| A5 | A(4,3,14) | -0.0382 | 0.6 |
| A8 | A(3,5,21) | 0.034 | 0.5 |
| A9 | A(6,5,21) | -0.039 | 0.6 |
| A11 | A(5,6,8) | -0.0301 | 0.5 |
| A12 | A(7,6,8) | 0.0492 | 0.8 |
| A14 | A(2,8,16) | -0.038 | 0.6 |
| A15 | A(6,8,16) | 0.0272 | 0.4 |
| A16 | A(2,10,11) | -0.4616 | 7.1 |
| A17 | A(2,10,12) | 0.0344 | 0.5 |
| A18 | A(11,10,12) | 0.4272 | 6.6 |
| A19 | A(10,12,13) | 0.1778 | 2.7 |
| A20 | A(10,12,17) | 0.1091 | 1.7 |
| A21 | A(13,12,17) | -0.2868 | 4.4 |
| A22 | A(9,13,12) | 0.1396 | 2.2 |
| A23 | A(9,13,16) | -0.0396 | 0.6 |
| A24 | A(12,13,16) | -0.1 | 1.5 |
| A26 | A(3,14,16) | -0.0314 | 0.5 |
| A27 | A(15,14,16) | 0.0329 | 0.5 |
| A28 | A(8,16,13) | -0.0252 | 0.4 |
| A31 | A(12,17,18) | -0.2996 | 4.6 |
| A32 | A(12,17,19) | 0.1432 | 2.2 |
| A33 | A(12,17,20) | 0.1432 | 2.2 |
| A34 | A(18,17,19) | 0.1073 | 1.7 |
| A35 | A(18,17,20) | 0.1073 | 1.7 |
| A36 | A(19,17,20) | -0.1932 | 3.0 |
| D38 | D(10,12,17,19) | -0.0316 | 0.5 |
| D39 | D(10,12,17,20) | 0.0316 | 0.5 |
| D41 | D(13,12,17,19) | -0.0316 | 0.5 |
| D42 | D(13,12,17,20) | 0.0316 | 0.5 |

----------------------------------------------------------------------------

Normal Mode 48

| Name | Definition | Value | Relative Weight (%) |
|---|---|---|---|
| R1 | R(1,2) | -0.9209 | 9.7 |
| R2 | R(2,8) | 0.339 | 3.6 |
| R3 | R(2,10) | 0.4641 | 4.9 |
| R5 | R(3,5) | -0.1191 | 1.3 |
| R6 | R(3,14) | 0.0889 | 0.9 |
| R10 | R(6,8) | 0.1299 | 1.4 |
| R11 | R(8,16) | -0.2404 | 2.5 |
| R12 | R(9,13) | -0.5983 | 6.3 |
| R14 | R(10,12) | -0.4366 | 4.6 |
| R15 | R(12,13) | 0.2733 | 2.9 |
| R16 | R(12,17) | 0.1487 | 1.6 |
| R17 | R(13,16) | 0.2666 | 2.8 |
| A1 | A(1,2,8) | 0.2274 | 2.4 |
| A2 | A(1,2,10) | 0.2301 | 2.4 |
| A3 | A(8,2,10) | -0.4575 | 4.8 |
| A4 | A(4,3,5) | 0.08 | 0.8 |
| A5 | A(4,3,14) | -0.0983 | 1.0 |
| A8 | A(3,5,21) | 0.0641 | 0.7 |
| A9 | A(6,5,21) | -0.1089 | 1.1 |
| A11 | A(5,6,8) | -0.0912 | 1.0 |
| A13 | A(2,8,6) | -0.2748 | 2.9 |
| A14 | A(2,8,16) | 0.2362 | 2.5 |
| A16 | A(2,10,11) | -0.5305 | 5.6 |
| A17 | A(2,10,12) | 0.2134 | 2.3 |
| A18 | A(11,10,12) | 0.3171 | 3.3 |
| A19 | A(10,12,13) | 0.2044 | 2.2 |
| A21 | A(13,12,17) | -0.2443 | 2.6 |
| A22 | A(9,13,12) | 0.1856 | 2.0 |

| | | | |
|---|---|---|---|
| A23 | A(9,13,16) | 0.1388 | 1.5 |
| A24 | A(12,13,16) | -0.3245 | 3.4 |
| A26 | A(3,14,16) | -0.0791 | 0.8 |
| A27 | A(15,14,16) | 0.0785 | 0.8 |
| A28 | A(8,16,13) | 0.1279 | 1.3 |
| A29 | A(8,16,14) | 0.0686 | 0.7 |
| A30 | A(13,16,14) | -0.1966 | 2.1 |
| A31 | A(12,17,18) | -0.1405 | 1.5 |
| A34 | A(18,17,19) | 0.0644 | 0.7 |
| A35 | A(18,17,20) | 0.0644 | 0.7 |
| A36 | A(19,17,20) | -0.0767 | 0.8 |

----------------------------------------------------------------------------

Normal Mode 49

| Name | Definition | Value | Relative Weight (%) |
|---|---|---|---|
| R1 | R(1,2) | -0.6982 | 8.0 |
| R2 | R(2,8) | 0.1563 | 1.8 |
| R3 | R(2,10) | 0.219 | 2.5 |
| R6 | R(3,14) | 0.1309 | 1.5 |
| R7 | R(5,6) | -0.1514 | 1.7 |
| R10 | R(6,8) | 0.2291 | 2.6 |
| R12 | R(9,13) | 1.0465 | 12.1 |
| R14 | R(10,12) | 0.0795 | 0.9 |
| R15 | R(12,13) | -0.3504 | 4.0 |
| R16 | R(12,17) | -0.092 | 1.1 |
| R17 | R(13,16) | -0.2928 | 3.4 |
| R19 | R(14,16) | -0.2545 | 2.9 |
| A1 | A(1,2,8) | 0.1718 | 2.0 |
| A2 | A(1,2,10) | 0.1296 | 1.5 |
| A3 | A(8,2,10) | -0.3015 | 3.5 |

| | | | |
|---|---|---|---|
| A6 | A(5,3,14) | -0.0826 | 1.0 |
| A7 | A(3,5,6) | 0.0727 | 0.8 |
| A8 | A(3,5,21) | -0.07 | 0.8 |
| A10 | A(5,6,7) | 0.1601 | 1.8 |
| A12 | A(7,6,8) | -0.1448 | 1.7 |
| A13 | A(2,8,6) | -0.1491 | 1.7 |
| A14 | A(2,8,16) | 0.243 | 2.8 |
| A15 | A(6,8,16) | -0.0939 | 1.1 |
| A16 | A(2,10,11) | -0.2052 | 2.4 |
| A17 | A(2,10,12) | 0.1491 | 1.7 |
| A18 | A(11,10,12) | 0.0561 | 0.6 |
| A19 | A(10,12,13) | -0.2395 | 2.8 |
| A21 | A(13,12,17) | 0.2519 | 2.9 |
| A22 | A(9,13,12) | -0.2221 | 2.6 |
| A23 | A(9,13,16) | -0.2458 | 2.8 |
| A24 | A(12,13,16) | 0.468 | 5.4 |
| A25 | A(3,14,15) | -0.1703 | 2.0 |
| A27 | A(15,14,16) | 0.1265 | 1.5 |
| A28 | A(8,16,13) | -0.3192 | 3.7 |
| A29 | A(8,16,14) | 0.0753 | 0.9 |
| A30 | A(13,16,14) | 0.2439 | 2.8 |
| A31 | A(12,17,18) | 0.1052 | 1.2 |
| A34 | A(18,17,19) | -0.0656 | 0.8 |
| A35 | A(18,17,20) | -0.0656 | 0.8 |

-----------------------------------------------------------------------------

Normal Mode 50

------------------------- -------------------------

| Name | Definition | Value | Relative Weight (%) |
|---|---|---|---|
| R16 | R(12,17) | 0.0489 | 2.5 |
| R20 | R(17,18) | -0.4926 | 24.9 |

| | | | |
|---|---|---|---|
| R21 | R(17,19) | -0.6397 | 32.4 |
| R22 | R(17,20) | -0.6397 | 32.4 |

Normal Mode 51

| Name | Definition | Value | Relative Weight (%) |
|---|---|---|---|
| R21 | R(17,19) | -0.7768 | 37.7 |
| R22 | R(17,20) | 0.7768 | 37.7 |
| A32 | A(12,17,19) | 0.0385 | 1.9 |
| A33 | A(12,17,20) | -0.0385 | 1.9 |
| A34 | A(18,17,19) | 0.0395 | 1.9 |
| A35 | A(18,17,20) | -0.0395 | 1.9 |
| D30 | D(2,10,12,17) | -0.0383 | 1.9 |
| D32 | D(11,10,12,17) | -0.0386 | 1.9 |
| D35 | D(17,12,13,9) | 0.0354 | 1.7 |
| D36 | D(17,12,13,16) | 0.0358 | 1.7 |
| D37 | D(10,12,17,18) | 0.0725 | 3.5 |
| D40 | D(13,12,17,18) | 0.036 | 1.7 |
| D41 | D(13,12,17,19) | -0.0402 | 2.0 |
| D42 | D(13,12,17,20) | -0.0402 | 2.0 |

Normal Mode 52

| Name | Definition | Value | Relative Weight (%) |
|---|---|---|---|
| R13 | R(10,11) | -0.0434 | 2.0 |
| R20 | R(17,18) | 0.9625 | 43.4 |
| R21 | R(17,19) | -0.38 | 17.1 |
| R22 | R(17,20) | -0.38 | 17.1 |
| A20 | A(10,12,17) | 0.0314 | 1.4 |

| | | | |
|---|---|---|---|
| A21 | A(13,12,17) | -0.0321 | 1.4 |
| A31 | A(12,17,18) | -0.0474 | 2.1 |
| A32 | A(12,17,19) | 0.028 | 1.3 |
| A33 | A(12,17,20) | 0.028 | 1.3 |
| A36 | A(19,17,20) | 0.0414 | 1.9 |
| D38 | D(10,12,17,19) | 0.0424 | 1.9 |
| D39 | D(10,12,17,20) | -0.0424 | 1.9 |
| D41 | D(13,12,17,19) | 0.0424 | 1.9 |
| D42 | D(13,12,17,20) | -0.0424 | 1.9 |

---------------------------------------------------------------------------

Normal Mode 53

| Name | Definition | Value | Relative Weight (%) |
|---|---|---|---|
| R3 | R(2,10) | -0.0372 | 2.4 |
| R13 | R(10,11) | 1.0821 | 69.8 |
| R14 | R(10,12) | -0.0517 | 3.3 |
| R20 | R(17,18) | 0.0442 | 2.9 |
| A2 | A(1,2,10) | 0.0287 | 1.9 |
| A3 | A(8,2,10) | -0.0272 | 1.8 |
| A16 | A(2,10,11) | -0.0264 | 1.7 |
| A17 | A(2,10,12) | 0.0567 | 3.7 |
| A18 | A(11,10,12) | -0.0303 | 2.0 |
| A19 | A(10,12,13) | -0.0272 | 1.8 |
| A20 | A(10,12,17) | 0.0311 | 2.0 |

---------------------------------------------------------------------------

Normal Mode 54

| Name | Definition | Value | Relative Weight (%) |
|---|---|---|---|
| R4 | R(3,4) | 0.7377 | 31.4 |

| | | | |
|---|---|---|---|
| R6 | R(3,14) | -0.0237 | 1.0 |
| R7 | R(5,6) | 0.0236 | 1.0 |
| R8 | R(5,21) | -0.7405 | 31.6 |
| R9 | R(6,7) | 0.1973 | 8.4 |
| R13 | R(10,11) | 0.0205 | 0.9 |
| R18 | R(14,15) | -0.1929 | 8.2 |
| A4 | A(4,3,5) | -0.0402 | 1.7 |
| A5 | A(4,3,14) | -0.0226 | 1.0 |
| A6 | A(5,3,14) | 0.0627 | 2.7 |
| A7 | A(3,5,6) | -0.0629 | 2.7 |
| A8 | A(3,5,21) | 0.0402 | 1.7 |
| A9 | A(6,5,21) | 0.0228 | 1.0 |
| A10 | A(5,6,7) | -0.0265 | 1.1 |
| A11 | A(5,6,8) | 0.0288 | 1.2 |
| A25 | A(3,14,15) | 0.0263 | 1.1 |
| A26 | A(3,14,16) | -0.0285 | 1.2 |

---------------------------------------------------------------------------

Normal Mode 55

| Name | Definition | Value | Relative Weight (%) |
|---|---|---|---|
| R4 | R(3,4) | -0.6945 | 28.2 |
| R5 | R(3,5) | 0.0603 | 2.4 |
| R8 | R(5,21) | -0.6876 | 27.9 |
| R9 | R(6,7) | 0.3349 | 13.6 |
| R18 | R(14,15) | 0.3258 | 13.2 |
| A10 | A(5,6,7) | -0.0316 | 1.3 |
| A11 | A(5,6,8) | 0.0358 | 1.5 |
| A25 | A(3,14,15) | -0.0314 | 1.3 |
| A26 | A(3,14,16) | 0.0355 | 1.4 |

---------------------------------------------------------------------------

Normal Mode 56

| Name | Definition | Value | Relative Weight (%) |
|---|---|---|---|
| R4 | R(3,4) | -0.1545 | 6.7 |
| R6 | R(3,14) | 0.0367 | 1.6 |
| R7 | R(5,6) | -0.0466 | 2.0 |
| R8 | R(5,21) | 0.2347 | 10.1 |
| R9 | R(6,7) | 0.8083 | 34.9 |
| R10 | R(6,8) | -0.0332 | 1.4 |
| R18 | R(14,15) | -0.6649 | 28.7 |
| R19 | R(14,16) | 0.0274 | 1.2 |
| A4 | A(4,3,5) | 0.0147 | 0.6 |
| A5 | A(4,3,14) | -0.0167 | 0.7 |
| A8 | A(3,5,21) | -0.0149 | 0.6 |
| A9 | A(6,5,21) | 0.0184 | 0.8 |
| A10 | A(5,6,7) | -0.0187 | 0.8 |
| A11 | A(5,6,8) | 0.0372 | 1.6 |
| A12 | A(7,6,8) | -0.0185 | 0.8 |
| A13 | A(2,8,6) | 0.0228 | 1.0 |
| A15 | A(6,8,16) | -0.0208 | 0.9 |
| A25 | A(3,14,15) | 0.0165 | 0.7 |
| A26 | A(3,14,16) | -0.0317 | 1.4 |
| A27 | A(15,14,16) | 0.0152 | 0.7 |
| A29 | A(8,16,14) | 0.0168 | 0.7 |
| A30 | A(13,16,14) | -0.0187 | 0.8 |

Normal Mode 57

| Name | Definition | Value | Relative Weight (%) |
|---|---|---|---|

| | | | |
|---|---|---|---|
| R4 | R(3,4) | -0.348 | 14.2 |
| R5 | R(3,5) | 0.0264 | 1.1 |
| R6 | R(3,14) | 0.0488 | 2.0 |
| R7 | R(5,6) | 0.04 | 1.6 |
| R8 | R(5,21) | -0.3089 | 12.6 |
| R9 | R(6,7) | -0.6087 | 24.8 |
| R10 | R(6,8) | 0.0244 | 1.0 |
| R18 | R(14,15) | -0.7682 | 31.4 |
| R19 | R(14,16) | 0.031 | 1.3 |
| A11 | A(5,6,8) | -0.0238 | 1.0 |
| A13 | A(2,8,6) | -0.0169 | 0.7 |
| A15 | A(6,8,16) | 0.0165 | 0.7 |
| A26 | A(3,14,16) | -0.0313 | 1.3 |
| A27 | A(15,14,16) | 0.0183 | 0.7 |
| A29 | A(8,16,14) | 0.0206 | 0.8 |
| A30 | A(13,16,14) | -0.0214 | 0.9 |
